# Structure of DNMT3B homo-oligomer reveals vulnerability to impairment by ICF mutations

**DOI:** 10.1038/s41467-022-31933-w

**Published:** 2022-07-22

**Authors:** Linfeng Gao, Yiran Guo, Mahamaya Biswal, Jiuwei Lu, Jiekai Yin, Jian Fang, Xinyi Chen, Zengyu Shao, Mengjiang Huang, Yinsheng Wang, Gang Greg Wang, Jikui Song

**Affiliations:** 1 Environmental Toxicology Graduate Program, University of California, Riverside, 92521 CA USA; 2 Lineberger Comprehensive Cancer Center, University of North Carolina at Chapel Hill School of Medicine, Chapel Hill, 27599 NC USA; 3 Curriculum in Genetics and Molecular Biology, University of North Carolina at Chapel Hill, Chapel Hill, 27599 NC USA; 4 Department of Biochemistry, University of California, Riverside, 92521 CA USA; 5 Department of Chemistry, University of California, Riverside, 92521 CA USA; 6 Department of Biochemistry and Biophysics, University of North Carolina at Chapel Hill School of Medicine, Chapel Hill, 27599 NC USA; 7 Department of Pharmacology, University of North Carolina at Chapel Hill School of Medicine, Chapel Hill, 27599 NC USA

**Keywords:** DNA methylation, X-ray crystallography, DNA methylation

## Abstract

DNA methyltransferase DNMT3B plays an essential role in establishment of DNA methylation during embryogenesis. Mutations of DNMT3B are associated with human diseases, notably the immunodeficiency, centromeric instability and facial anomalies (ICF) syndrome. How ICF mutations affect DNMT3B activity is not fully understood. Here we report the homo-oligomeric structure of DNMT3B methyltransferase domain, providing insight into DNMT3B-mediated DNA methylation in embryonic stem cells where the functional regulator DNMT3L is dispensable. The interplay between one of the oligomer interfaces (FF interface) and the catalytic loop renders DNMT3B homo-oligomer a conformation and activity distinct from the DNMT3B-DNMT3L heterotetramer, and a greater vulnerability to certain ICF mutations. Biochemical and cellular analyses further reveal that the ICF mutations of FF interface impair the DNA binding and heterochromatin targeting of DNMT3B, leading to reduced DNA methylation in cells. Together, this study provides a mechanistic understanding of DNMT3B-mediated DNA methylation and its dysregulation in disease.

## Abstract (web summary)

In mammals, de novo DNA methyltransferase DNMT3B is essential for establishing DNA methylation patterns during embryonic development. Here the authors provide a structure-function characterization of homo-oligomeric DNMT3B, revealing the molecular basis for its vulnerability to ICF mutations.

## Introduction

DNA methylation is an important epigenetic mechanism that is essential for gene regulation and genomic stability^1^. In mammals, DNA methylation is established by two closely-related de novo DNA methyltransferases, DNMT3A and DNMT3B, which play overlapping but distinct roles in orchestrating DNA methylation patterns during development^2–4^. DNMT3A-mediated DNA methylation in germ cells critically depends on the functional regulator DNMT3L^5–8^. Previous structural studies of DNMT3A in complex with DNMT3L reveal a heterotetrameric assembly with two alternative interfaces: a hydrophilic interface (a.k.a. RD interface) mediates DNMT3A homodimerzation, while a hydrophobic interface (a.k.a. FF interface) mediates the heterodimerzation between DNMT3A and DNMT3L^9^. The two interfaces are not only important for DNMT3A activity, but also critical for heterochromatic targeting of DNMT3A^10^. On the other hand, although DNMT3L also binds and stimulates DNMT3B in vitro, it is dispensable for DNMT3B-mediated DNA methylation in embryonic stem (ES) cells^6,11–14^. This observation suggests a DNMT3L-independent activity of DNMT3B.

The Immunodeficiency, centromeric instability and facial anomalies (ICF) syndrome is an autosomal recessive disease characterized by immunodeficiency, developmental delay and facial dysmorphism^15^. The centromeric instability of this disease arises from DNA hypomethylation at pericentromeric satellite repeats^16^. Previous studies have linked ICF syndrome to homozygous or compound heterozygous mutations of DNMT3B, occurring in approximately half of the patients with ICF syndrome^4,17,18^. The majority of the ICF-associated DNMT3B mutations are localized to the C-terminal methyltransferase (MTase) domain^19^, manifesting compromised DNA methylation activity^19–21^. These observations suggest an intricate link between the enzymatic activity of DNMT3B and pathogenesis of ICF syndrome.

The crystal structures of DNMT3B in complex with DNMT3L, either in the presence or absence of DNA substrates, have recently been reported^22,23^. The DNMT3B-DNMT3L complex shows high structural resemblance with the DNMT3A-DNMT3L complex^9,24–26^, both present in a heterotetrameric form, in which DNMT3A or DNMT3B homodimerizes via the RD interface and each DNMT3A or DNMT3B monomer further associates with a DNMT3L molecule via the FF interface. Nevertheless, detailed structural comparison of the two complexes revealed subtle yet functionally important differences in substrate recognition, with the RD interface of DNMT3A engaging stronger DNA contacts than that of DNMT3B^22,23,26^. Furthermore, the DNA recognition loops in the target recognition domain (TRD) of DNMT3A and DNMT3B bind to the CpG sites in a distinct, flanking sequence-dependent manner, which gives rise to the strong, differential preferences of DNMT3A and DNMT3B toward a pyrimidine and purine at the +1 CpG-flanking site, respectively^22^. However, due to the lack of a structure of DNMT3B homo-oligomer, the molecular basis for DNMT3B-mediated DNA methylation in ES cells remains far from clear.

To characterize the structural underpinning of the DNMT3B-mediated DNA methylation and its dysregulation in human diseases, we determined the crystal structure of homo-oligomeric DNMT3B methyltransferase (MTase) domain. Structural comparison of DNMT3B homo-oligomer with the DNMT3B-DNMT3L heterotetramer reveals distinct intermolecular interactions at the FF interface, resulting in different conformation and activity of DNMT3B. The ICF mutations on the FF interface, R670Q and L664P/L664T, lead to more pronounced disruption of the DNMT3B oligomer than to the DNMT3B-DNMT3L complex. Through biochemical and cellular analysis, we further show that these ICF mutations at the FF interface impair the DNA binding and heterochromatin association of DNMT3B, leading to reduced DNA methylation in cells. Taken together, this study establishes an etiological role of DNMT3B oligomerization in ICF syndrome.

## Results

### Crystal structure of the homo-oligomeric complex of DNMT3B MTase domain

**Fig. 1:**
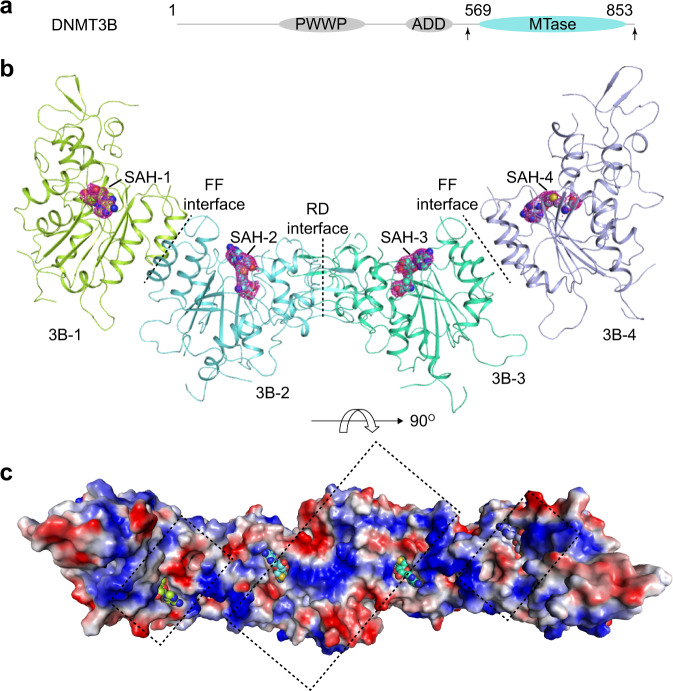
Crystal structure of the homo-oligomeric DNMT3B MTase domains. **a** Color-coded domain architecture of DNMT3B, with the MTase domain used for structural study delimited by arrows. Ribbon diagram (**b**) and electrostatic surface (**c**) of the tetrameric unit of DNMT3B homo-oligomer, with the DNMT3B-bound SAH molecules shown in sphere representation. The individual DNMT3B molecules are labeled as 3B-1, 3B-2, 3B-3, and 3B-4, respectively. The potential DNA-binding surface of the RD interface-mediated DNMT3B dimer is indicated by a rectangle. In the electrostatic surface, red, blue and grey colors indicate negative, positive and neutral potential, respectively.

To investigate the structural basis underlying DNA methylation by DNMT3B homo-oligomer, we crystalized the DNMT3B MTase domain in the presence of *S*-adenosyl-homocysteine (SAH), a byproduct of methyl-donor *S*-adenosyl-methionine (SAM), and solved the structure at 3.27 Å resolution (Fig. 1a, b and Supplementary Table 1). The crystal structure of SAH-bound DNMT3B MTase domain belongs to the space group of P3_2_. There are two DNMT3B tetrameric assemblies in each asymmetric unit, arranged in a parallel fashion (Supplementary Fig. 1a). Each DNMT3B monomer is bound to one SAH molecule (Fig. 1b and Supplementary Fig. 1a). The tetrameric assembly of DNMT3B MTase domain (hereinafter referred to as DNMT3B-DNMT3B complex) is reminiscent of that of the DNMT3B-DNMT3L tetramer, with the two DNMT3B monomers associated via the RD interface to form a continuous substrate-binding interface involving the TRD, catalytic core and the RD interface (Fig. 1b, c). Two additional DNMT3B monomers are attached to the RD interface-mediated DNMT3B dimer through the FF interfaces, with their potential DNA-binding surface positioned separately from the two RD interface-related DNMT3B monomers (Fig. 1b).

It is worth noting that the crystal structure of DNMT3B in fact illustrates a macro-oligomeric assembly: the presence of FF and RD interfaces permits the DNMT3B molecules to pack into a left-handed helical structure in crystals, in a periodicity of DNMT3B decamer (Supplementary Fig. 1b). The functional implication of this supramolecular assembly remains unclear and subject to investigation in future. Nevertheless, for the purpose of comparison with the DNMT3B-DNMT3L complex, we focus on discussing the tetrameric unit of DNMT3B macro-oligomer in this study.

### Structural comparison of DNMT3B-DNMT3B and DNMT3B-DNMT3L complexes

**Fig. 2:**
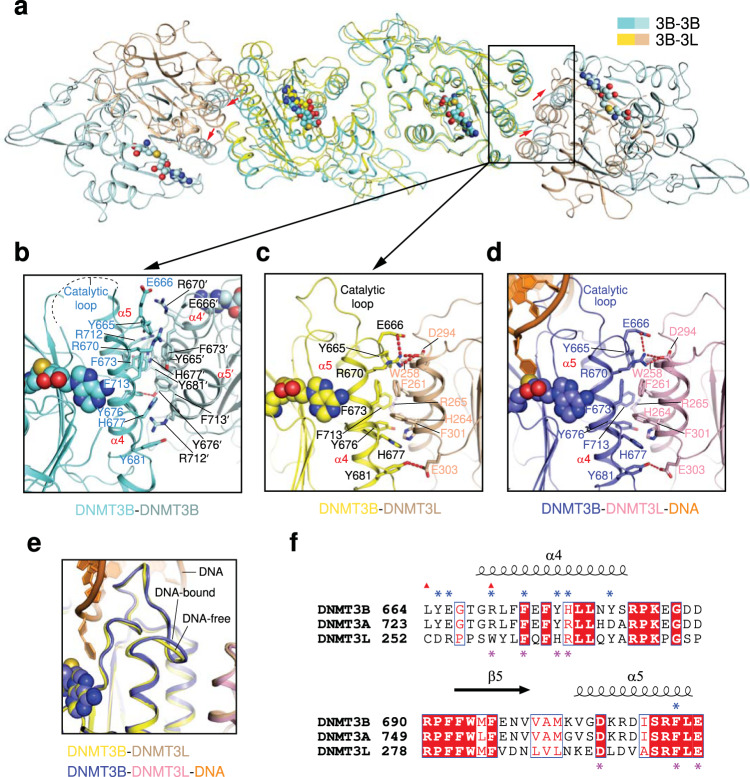
Structural comparison between DNMT3B homo-oligomer and the DNMT3B-DNMT3L complex. **a** Structural overlay of the tetrameric unit of DNMT3B homo-oligomer (aquamarine and light blue) and the DNMT3B (yellow)-DNMT3L (wheat) (PDB 6KDP) complexes. 3B: DNMT3B, 3L: DNMT3L. Close-up view of the FF interface and the catalytic loop of DNMT3B in the DNMT3B homo-oligomer (**b**) and the DNMT3B-DNMT3L heterotetramer (**c**) that are overlaid in **a**. In **b** the disordered catalytic loop is shown as a dashed line. Residues from the symmetry-related DNMT3B monomer are indicated by prime (′). **d** Close-up view of the FF interface and the catalytic loop of DNMT3B in DNA (orange)-bound DNMT3B (slate)-DNMT3L (light pink) complex (PDB 6U8P). **e** Close-up view of the catalytic loop aligned between DNA-free and DNA-bound DNMT3B-DNMT3L complexes. **f** Structure-based sequence alignment of human DNMT3B, DNMT3A and DNMT3L. Identical residues are colored white in red background. Similar residues are colored red. The ICF mutations are marked by red triangles on top of the sequences. The residues involved in the intermolecular interactions at the FF interfaces of DNMT3B and DNMT3L are marked by blue and purple asterisks, on top and at the bottom of the sequences, respectively.

To elaborate how the different oligomeric states of DNMT3B affect its activity, we next performed structural comparison between the DNMT3B-DNMT3B complex and the DNMT3B-DNMT3L complex, either free or the DNA-bound form. Superposition of the two RD interface-related DNMT3B monomers of the DNMT3B-DNMT3B complex with those of DNMT3B-DNMT3L (PDB 6KDP) and DNMT3B-DNMT3L-DNA (PDB 6U8P) complexes gives a root-mean-square deviation (RMSD) of 1.0 Å and 0.97 Å over 517 and 532 aligned Cα atoms, respectively, indicative of high structural similarity between these complexes (Fig. 2a and Supplementary Fig. 2a). In all three complexes, DNMT3B presents the same set of residues to form the RD interface (Supplementary Fig. 2b–d), as well as the SAH-binding pocket (Supplementary Fig. 2e–g). Furthermore, the FF interfaces of these complexes are similarly mediated by a helical bundle: in the DNMT3B-DNMT3B complex, the two FF interface-related DNMT3B monomers each present one pair of α-helices (α4 and α5 in DNMT3B) for helical packing, resembling the four helix-bundle formed in the DNMT3B-DNMT3L complexes (Fig. 2a–d and Supplementary Fig. 2a). Of note, the FF interface in the DNMT3B-DNMT3L complexes is dominated by a hydrophobic cluster formed by DNMT3B Y665, F673, Y676, H677, Y681 and F713 and DNMT3L W258, F261, H264 and F301 (Fig. 2c, d). In addition, a set of hydrogen-bonding/cation-π interactions involving DNMT3B R670, Y681 and F713 and DNMT3L R265, D294 and E303 reinforce the DNMT3B-DNMT3L association (Fig. 2c, d). Likewise, a similar set of DNMT3B residues engage in the formation of hydrophobic cluster within the FF interface of the DNMT3B-DNMT3B complex (Fig. 2b).

Notably, the DNA-interacting loop from the TRD was poorly traced in both the DNA-free DNMT3B-DNMT3B and DNMT3B-DNMT3L complexes (Supplementary Fig. 2h, i), but adopts a well-defined conformation in the DNMT3B-DNMT3L-DNA complex (Supplementary Fig. 2j), supporting the notion that this segment undergoes a disorder-to-order transition upon DNA binding. Another notable structural difference between the DNMT3B-DNMT3B and DNMT3B-DNMT3L complexes lies in the relative positioning of the interacting helices at the FF interface, which undergoes a lateral shift of 6-7 Å between the two complexes (Fig. 2a, see red arrows). Near to the FF interface, the loop harboring the catalytic cysteine C657 (denoted as catalytic loop herein) also shows large conformational difference: Residues N652-R661 of the catalytic loop are untraceable in the DNMT3B-DNMT3B complex but adopt a preconfigured conformation for DNA binding in the DNMT3B-DNMT3L complex (Fig. 2e).

### The FF interface modulates the catalytic loop conformation of DNMT3B

Detailed structural comparison of the DNMT3B-DNMT3B and DNMT3B-DNMT3L complexes reveals a difference in the intermolecular interactions at the FF interface (Fig. 2b–d). In the DNMT3B-DNMT3L complexes, DNMT3B R670 on the α-helix (α4) that immediately follows the catalytic loop stabilizes the FF interface via salt bridge interactions with DNMT3B E666 and DNMT3L D294 (Fig. 2c, d). In the DNMT3B-DNMT3B complex, these interactions are replaced by reciprocal cation-π interactions between DNMT3B R670 of one monomer and DNMT3B Y665 of the other (Fig. 2b). As a result, the side chain of DNMT3B E666 flips away from R670 to become solvent exposed in the DNMT3B-DNMT3B complex (Fig. 2b), which likely contributes to the dynamic disorder of the catalytic loop (Fig. 2b). At the C-terminal end of α4-helix, the hydrogen bond formed by DNMT3B Y681 and DNMT3L E303 in the DNMT3B-DNMT3L complex becomes lost in the DNMT3B-DNMT3B complex, accompanied by the introduction of a side-chain hydrogen bond between the two DNMT3B Y676 residues from the two symmetry-related DNMT3B monomers, respectively (Fig. 2b–d).

The structural changes described above are accompanied by a lateral shift of the FF interfaces between the DNMT3B-DNMT3B and DNMT3B-DNMT3L complexes (Fig. 2a). As a result, the buried surface area at the FF interface is reduced from ~880 Å^2^ for the DNMT3B-DNMT3L complex to ~716 Å^2^ for the DNMT3B-DNMT3B complex, implying differential assembly stability between the two complexes. Indeed, our size-exclusion chromatography analysis of DNMT3B protein mixed with DNMT3L protein indicates that DNMT3B associates with DNMT3L more strongly than with other DNMT3B molecules (Supplementary Fig. 2k, l). Furthermore, considering that the catalytic loop of DNMT3B is responsible for accessing the DNA minor groove and anchoring the target cytosine during catalysis (Fig. 2d), the differential conformational dynamics of the catalytic loop between the DNMT3B-DNMT3B and DNMT3B-DNMT3L complexes provides an explanation for the enzymatic stimulation of DNMT3A and DNMT3B by DNMT3L. It remains to be investigated whether and how the conformation of the FF interface of DNMT3B differs among various homo-oligomeric (e.g. macro-oligomeric vs tetrameric) states.

Sequence analysis of the residues involved in the formation of the FF interface indicates that most of these residues are conserved between DNMT3B and DNMT3A (Fig. 2f). The exceptions lie in DNMT3B H677 and Y681, which are replaced by an arginine (R736) and an aspartate (D740), respectively, in DNMT3A (Fig. 2f), suggesting similar but distinct interaction mechanisms underpinning the homo-oligomeric states between DNMT3A and DNMT3B. On the other hand, the sequences of DNMT3A/DNMT3B and DNMT3L show a more substantial difference. Notably, a tyrosine in the DNMT3B Y665-corresponding site of DNMT3A and DNMT3B is replaced by an aspartate (D253) in DNMT3L, a glutamate in the DNMT3B E666-corresponding site of DNMT3A and DNMT3B is replaced by an arginine (R254) in DNMT3L, an arginine in the DNMT3B R670-corresponding site of DNMT3A and DNMT3B is replaced by a tryptophan (W258) in DNMT3L, and a tyrosine in the DNMT3B Y676-corresponding site of DNMT3A and DNMT3B is replaced by a histidine (H264) in DNMT3L (Fig. 2f). These sequence variations between DNMT3B and DNMT3L underlie the different assembly stabilities between the DNMT3B-DNMT3B and DNMT3B-DNMT3L complexes.

### Structural mapping of the DNMT3B ICF mutations

To understand the functional consequence of the ICF-associated DNMT3B mutations, we mapped the reported ICF mutations to the structures of the DNMT3B homo-oligomer and our previously reported DNMT3B-DNMT3L-DNA complex (Supplementary Fig. 3). It is apparent that the ICF mutations spread over several functionally distinct regions, including the structural core, the SAM- and DNA-binding sites, the RD interface, and the FF interface (Supplementary Fig. 3). Among these, the A603T, V726G, A766P and R840Q mutations located in the structural core reportedly reduced the activity and/or oligomerization of DNMT3B, likely due to an impact on the protein stability^20,21,27^. The ICF mutations associated with the DNA binding (e.g. G663S and R823G) or SAM binding (e.g. V606A) have also been examined previously, which indicated that these mutations led to reduced enzymatic activity in the context of both DNMT3B-DNMT3B and the DNMT3B-DNMT3L complexes^19–22^. In addition, the mutations on the RD interface, H814R, have been shown to impair the DNMT3B activity via disruption of protein oligomerization^21^.

**Fig. 3:**
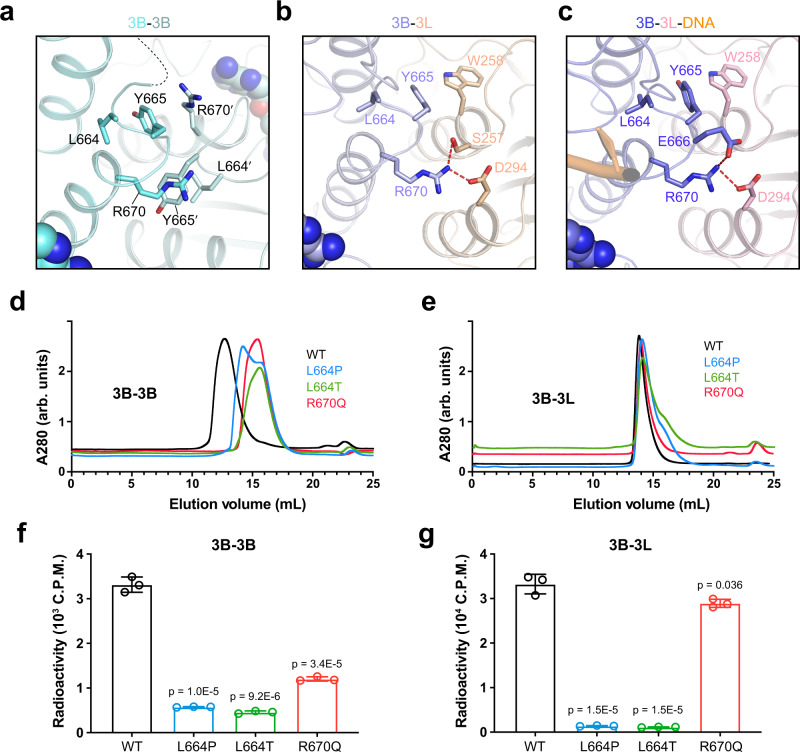
Structural and biochemical analysis of the ICF mutations on the FF interface. Close-up view the ICF-associated mutation sites in the DNMT3B-DNMT3B (**a**) DNMT3B-DNMT3L (**b**) and DNMT3B-DNMT3L-DNA (**c**) complexes. The color scheme is the same as that in Fig. 2a. Size-exclusion chromatography analysis of the DNMT3B-DNMT3B (**d**) and DNMT3B-DNMT3L (**e**) complexes harboring the ICF mutations on the FF interface. The MTase domain of DNMT3B and the C-terminal domain of DNMT3L were used for the analysis. In vitro DNA methylation assays of the DNMT3B-DNMT3B (**f**) and DNMT3B-DNMT3L (**g**) complexes harboring the ICF mutations on the FF interface. Data are mean±s.d. (*n* = 3 biological replicates). The two-tailed Student *t*-tests were performed to compare WT vs mutant. Source data are provided as a Source Data file.

The FF interface of DNMT3B contains at least two ICF mutation sites, L664 and R670 (Supplementary Fig. 3d, h), with L664 mutated to proline (L664P) or threonine (L664T) and R670 to glutamine (R670Q)^18,28,29^. Structural analysis of the DNMT3B-DNMT3L and DNMT3B-DNMT3B complexes reveals that L664 is located at the C-terminal end of the catalytic loop, while R670 is located on the subsequent α4-helix (Fig. 2b–d). As described above, DNMT3B R670 is directly involved in the FF interface in both the DNMT3B-DNMT3B and DNMT3B-DNMT3L complexes (Fig. 2b–d). However, it adopts different interaction mechanisms between these complexes, contacting DNMT3L D294 in the DNMT3B-DNMT3L complexes but DNMT3B Y665 in the DNMT3B-DNMT3B complex (Fig. 3a–c). On the other hand, DNMT3B L664 engages in a sidechain stacking interaction with the immediate downstream residue Y665 in both DNMT3B-DNMT3L and DNMT3B-DNMT3B complexes (Fig. 3a–c), which presumably stabilizes the conformation of both the FF interface and the catalytic loop. These observations suggest that both R670- and L664-associated mutations may affect the oligomeric assembly of DNMT3B, while the L664-associated mutations may further generate a direct impact on the conformation of the catalytic loop.

### ICF mutations of FF interface impact DNMT3B-DNMT3B and DNMT3B-DNMT3L differentially

Next, we performed size-exclusion chromatography to examine the effects of the FF interface-associated ICF mutations on the assembly of DNMT3B complexes. Wild-type (WT) DNMT3B MTase domain alone eluted at a volume that corresponds to an oligomeric form (tetramer or higher-order oligomer; Fig. 3d and Supplementary Fig. 4). Likewise, WT DNMT3B MTase in complex with the C-terminal domain of DNMT3L eluted at a volume that largely matches with its tetrameric form (Fig. 3e and Supplementary Fig. 4). Note that the elution peak for the DNMT3B homo-oligomer appears much broader than that of the DNMT3B-DNMT3L complex, likely due to the dynamic exchange between different oligomeric states of DNMT3B.

Size-exclusion chromatography analysis of the FF interface-associated ICF mutants further reveals that the L664P, L664T and R670Q mutations all led to pronounced delay of the DNMT3B-DNMT3B migration, with elution profiles corresponding to the dimeric and/or monomeric forms of DNMT3B (Fig. 3d). These observations confirm that the FF interface-associated ICF mutations impair the assembly of the DNMT3B-DNMT3B complex. Interestingly, a much weaker effect of the ICF mutations was observed on the DNMT3B-DNMT3L complex: in comparison with WT DNMT3B-DNMT3L, the L664P and L664T mutations only led to slight appearance of a trailing peak, while the R670Q mutation did not cause any appreciable change of the elution profile (Fig. 3e). This differential impact of the ICF mutations on the DNMT3B-DNMT3B and DNMT3B-DNMT3L complexes may be attributed to the different extent of intermolecular interactions at the FF interface of these two complexes: The FF interface in the DNMT3B-DNMT3L complex, in accordance with its larger buried surface area, is less susceptible to a disruption by the ICF mutations than that in the DNMT3B-DNMT3B complex.

We further performed in vitro DNA methylation assays for the DNMT3B-DNMT3B and DNMT3B-DNMT3L complexes on CpG DNA. First, the L664P, L664T and R670Q mutations led to 3-6-fold reduction of the methylation efficiency of the DNMT3B-DNMT3B complex. This observation, consistent with what was previously observed for the L664T mutation^19,20^, supports the notion that these mutations lead to the impairment of the FF interface of the DNMT3B-DNMT3B complex (Fig. 3f). Second, the L664P and L664T mutations also severely impaired the activity of the DNMT3B-DNMT3L complex (Fig. 3g), despite that they only led to a modest effect on the oligomerization of the DNMT3B-DNMT3L complex (Fig. 3e). Considering that the sidechain interaction of DNMT3B L664 and Y665 serves to configure the C-terminal end of the catalytic loop (Fig. 3a–c), this observation suggests that the L664-associated ICF mutations affect the activity of DNMT3B-DNMT3L most likely through their influence on the conformation of the catalytic loop. Interestingly, we observed that the R670Q mutation only led to a ~20% reduction of methylation efficiency of DNMT3B-DNMT3L (Fig. 3g), in contrast to a ~3-fold reduction of methylation efficiency of the DNMT3B-DNMT3B complex by this mutation (Fig. 3f). This differential impact of the R670Q mutation on the DNMT3B-DNMT3B and DNMT3B-DNMT3L complexes further highlights the distinct nature of the FF interface between the two alternative complexes.

### Impairment of heterochromatin targeting of DNMT3B by the FF interface mutations

It has been demonstrated that both DNMT3A and DNMT3B are associated with pericentric heterochromatin in ES cells in a histone H3K9 trimethylation (H3K9me3)-dependent manner^30,31^. In addition, DNMT3B is recruited to methylate gene bodies via the interaction between its PWWP domain and histone H3 lysine 36 trimethylation (H3K36me3), which serves to prevent aberrant intragenic transcription^32,33^. The heterochromatic association of DNMT3A appears to be regulated by its oligomerization, as disruption of either the FF or the RD interface led to reduced chromatin association of DNMT3A^10^. Likewise, ICF mutation DNMT3B A603T, which disrupted the DNMT3A-DNMT3B heterodimerization and DNMT3B homodimerization, has been shown to cause impaired heterochromatin association of DNMT3B^27^.

To evaluate the effect of the ICF mutations of the FF interface on the chromatin association of DNMT3B, we first performed ITC binding assays for WT and mutant DNMT3B on a 24-mer DNA duplex containing multiple CpG sites. WT DNMT3B binds to DNA strongly, with a dissociation constant (*K*_d_) of 4 μM (Supplementary Fig. 5a). In contrast, the L664T- and R670Q-mutated DNMT3B proteins both show much reduced DNA binding under the experimental condition (Supplementary Fig. 5b, c). A consistent result was observed using the EMSA assay (Supplementary Fig. 5d). These observations suggest a link between the integrity of the FF interface and the DNA binding activity of DNMT3B.

**Fig. 4:**
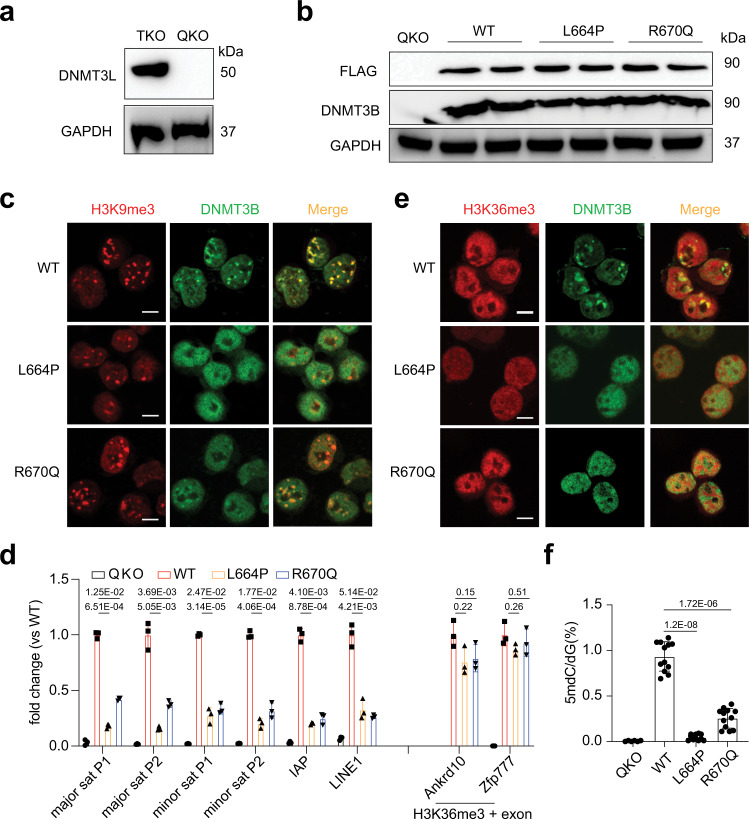
Cellular analysis of the ICF mutations on the FF interface of DNMT3B. **a** Immunoblotting validation for *Dnmt3l* knockout in TKO mESCs, generating QKO cells. **b** Immunoblotting of Flag-tagged full-length DNMT3B, either WT or the indicated mutant, after its stable transduction into QKO mESCs. **c** IF analysis for co-localization of H3K9me3 (red) and DNMT3B (green), either WT or the indicated FF interface mutant, after its stable transduction into QKO mESCs. Scale bar, 5 μm. **d** ChIP-qPCR for the DNMT3B binding at the indicated genomic locus at either heterochromatic regions (left) or H3K36me3-demarcated exons of actively transcribed genes (right). Major sat and minor sat refer to major and minor satellite DNA, respectively, with each probed by two independent primer sets (P1 and P2). Y-axis shows the averaged fold-change in ChIP-qPCR signals after normalization to those of input and then to WT samples. Error bars are mean±s.d. (*n* = 3 independent experiments). Two-sided Student *t*-test was applied for statistics, with the *p*-value labeled on the top of each panel. **e** IF analysis for co-localization of H3K36me3 and DNMT3B, either WT or the indicated FF interface mutant, after its stable transduction into QKO mESCs. Scale bar, 5 μm. **f** Mass spectrometry-based quantification for global DNA methylation levels in QKO cells with stable expression of the indicated DNMT3B. Data are mean±s.d. (two technical replicates each for 3 biological replicates for QKO, and 6 biological replicates for WT, L664P and R670Q). Paired *t*-test was applied for statistics, with *p*-value labeled on the top of each panel. Source data are provided as a Source Data file.

We further investigated the effect of the FF-interface mutations on the nuclear localization of DNMT3B by immunofluorescence (IF) assay. To eliminate the interference of DNMT3L, we used CRISPR/cas9-based gene editing technology to knock out Dnmt3l in the previously established mouse ES cells (ESCs) with triple knockout of Dnmt1, Dnmt3a and Dnmt3b (TKO)^32^, producing stable ESC lines with Dnmt1/Dnmt3a/Dnmt3b/Dnmt3l quadruple knockout (QKO) (Fig. 4a and Supplementary 6a–c). We chose not to knockout another reported de novo DNA methyltransferase, Dnmt3c^33^, due to its low activity in ESCs^34^. Subsequently, we transduced Flag-tagged WT DNMT3B into the QKO ESCs (Fig. 4b). Size-exclusion chromatography analysis of the purified Flag-DNMT3B from the transduced QKO cells indicated the presence of both a dominant tetramer and a less populated macro-oligomer (Supplementary Fig. 7a–c). Furthermore, our immunofluorescence assay revealed strong co-localization between WT DNMT3B and H3K9me3 (Fig. 4c and Supplementary Fig 8a), consistent with a previous report^35^. In contrast, the transduced DNMT3B with an FF mutation (L664P or R670Q) was expressed as the same levels as WT (Fig. 4b) but demonstrated a rather diffuse nuclear pattern and a significantly reduced co-localization with H3K9me3 (Fig. 4c and Supplementary Fig. 8a). Consistently, chromatin immunoprecipitation followed by quantitative PCR (ChIP-qPCR) assay showed that WT DNMT3B bound the repeat elements at pericentric heterochromatin including major and minor satellite DNA, Intracisternal A-particle (IAP) transposons, and LINE-1 (Fig. 4d; left, red vs. black [with QKO as a background control]), in line with the previous observation that DNMT3B preserves DNA methylation in both major and minor satellites in Dnmt3a knockout mice^36^. In comparison with WT, DNMT3B carrying an ICF mutation at the FF interface (L664P or R670Q) displayed significantly reduced bindings to these heterochromatin regions (Fig. 4d, left, orange and blue vs. red), suggesting that the ICF mutations on the FF interface compromise the heterochromatin targeting of DNMT3B. In light of previous reports that the heterochromatic recruitment of DNMT3B is regulated by H3K9me3 writer SETDB1, H3K9me3 reader HP1α, and histone deacetylase HDAC2^30,37–39^, we further performed co-immunoprecipitation analysis to evaluate the effect of the ICF mutations on the association of DNMT3B with partners. We found that neither L664P or R670Q mutation impacted the association of DNMT3B with HP1α and HDAC2 (Supplementary Fig. 8b), suggesting that the reduced heterochromatin association of L664P- or R670Q-mutated DNMT3B is likely attributed to the change of other DNMT3B-chromatin interaction elements, such as DNMT3B oligomerization and/or its DNA binding. These observations reinforce the notion that the integrity of DNMT3B oligomer interfaces is essential for its heterochromatin targeting.

The interaction between DNMT3B PWWP domain and H3K36me3 was shown to mediate the recruitment of DNMT3B to the body regions of transcribed genes^40^. As expected, our IF analysis additionally confirmed the co-localization of WT DNMT3B with H3K36me3 (Fig. 4e, top). The L664P or R670Q mutations did not affect the DNMT3B-H3K36me3 interaction to an extent that they did to the DNMT3B-H3K9me3 association (Fig. 4e and Supplementary Fig. 8c). In agreement, ChIP-qPCR showed the comparable bindings of WT DNMT3B and its FF mutants (L664P or R670Q) at the examined exon regions known to be demarcated by H3K36me3 (Fig. 4d, right; orange and blue vs. red). Overall, these results point to context-dependent DNMT3B targeting mechanisms.

### Impairment of DNMT3B-mediated DNA methylation by the FF-interface mutations

DNA hypomethylation at pericentric heterochromatin is one of the prominent features of ICF syndrome^4,18^. To examine how the ICF mutations on the FF interface affect DNMT3B-mediated DNA methylation, we performed mass spectrometry-based quantification of genomic DNA methylation to measure the global DNA methylation in QKO or TKO mouse ESC cells transduced with exogenous DNMT3B. The collective removal of DNMT1, DNMT3A and DNMT3L led to a great reduction of DNA methylation even for cells transduced with WT DNMT3B (Fig. 4f, see QKO and WT). Nevertheless, relative to WT DNMT3B-expressing cells, those with the L664P mutant showed greatly reduced global cytosine methylation in QKO cells (Fig. 4f). A similar observation was made by using the TKO cells as the rescue system (Supplementary Fig. 8d). On the other hand, those with the R670Q mutation showed a more pronounced reduction of global DNA methylation in QKO cells than in TKO cells (Fig. 4f vs Supplementary Fig. 8d), in line with the mutational effects observed in vitro in the context of DNMT3B homo-oligomer or the DNMT3B-DNMT3L complex. Together, these findings substantiate the impaired DNA methylation by DNMT3B homo-oligomer due to the FF interface mutations found in a subset of ICF patients.

## Discussion

DNMT3A and DNMT3B, the two major de novo DNA methyltransferases responsible for establishment of mammalian DNA methylation, possess overlapped but distinct functionalities in development^2,4,22^. Hetero-oligomerization with DNMT3L critically influences DNMT3A-mediated methylation, but not the DNMT3B-mediated DNA methylation^7,41,42^, which highlights the importance of a homo-oligomeric state regarding DNMT3B activity. This study reports the structure of DNMT3B homo-oligomer, which provides a unique opportunity for the field to gain critical insights into the molecular mechanism by which DNMT3B homo-oligomers (e.g. macro-oligomer) regulate genome methylation. Our combined structural, biochemical and cellular analyses further reveal how a subset of DNMT3B mutations detected among ICF patients influence functionality of DNMT3B homo-oligomer. Therefore, the findings presented in this work improve current understanding as for the roles for DNMT3B in organismal development and its functional impairment in developmental syndromes such as ICF.

The structures of the DNMT3B-DNMT3L complexes have recently been reported, providing a framework for structure-function understanding of the ICF mutations located on the DNA- and cofactor-binding sites or RD interface^22,23^. However, functional characterization of ICF mutations on the FF interface has been challenging, partly due to the fact that the two distinctive DNMT3B-DNMT3B and DNMT3B-DNMT3L complexes give rise to distinct intermolecular interactions at the FF interfaces. This study, through structure determination of the DNMT3B homo-oligomer, provides a clear view as for how FF-interface mutations affect the activity of homo-oligomeric DNMT3B. First, in line with the critical role of the FF interface in the oligomeric assembly of DNMT3B, the ICF mutations on the FF interface, L664P/T and R670Q, lead to impairment of DNMT3B oligomerization. Second, we find that the ICF mutations on the FF interface are located around the C-terminal end of the catalytic loop, which may contribute to the fact that these mutations also led to a decrease in DNA binding activity. Furthermore, the ICF mutations on the FF interface disrupt the assembly of DNMT3B homotetramer more pronouncedly than they do to the DNMT3B-DNMT3L heterotetramer, consistent with the fact that there is a differential dependency on intermolecular contacts seen at distinctive FF interfaces of DNMT3B-DNMT3B and DNMT3B-DNMT3L complexes. Strikingly, the ICF R670Q mutation causes a substantial activity loss of the DNMT3B-DNMT3B complex but a rather modest impairment on the DNMT3B-DNMT3L complex, in line with the fact that R670 engages in a sidechain stacking interaction in DNMT3B homo-oligomer but salt-bridge interactions in the DNMT3B-DNMT3L complex. This study therefore suggests that homo-oligomeric DNMT3B is more vulnerable to the impairment of ICF mutations than the DNMT3B-DNMT3L complex. A deeper understanding of DNMT3B and its disease-related mutations shall ultimately aid in the development of therapeutical strategies.

Additionally, previous studies demonstrated that the DNA methylation and SAM-binding activities of DNMT3A and DNMT3B can both be stimulated by DNMT3L in vitro^6,11–13^. However, the mechanism has not been well elucidated. In this study, we find that the transition from DNMT3B homo-oligomer to the DNMT3B-DNMT3L heterotetramer leads to more extensive intermolecular contacts at the FF interaction, as well as structural ordering of the catalytic loop, which provides an explanation to the enzymatic stimulation of DNMT3A and DNMT3B by DNMT3L.

Targeting DNMT3A and DNMT3B to heterochromatin is critical for DNA methylation at heterochromatic regions such as pericentric ones^30,37^. Although it has been established that heterochromatin recruitment of DNMT3A and DNMT3B is mediated by their interactions with heterochromatin-specific factors, such as SETDB1/HP1^30,37–39^, how the oligomerization of DNMT3A and DNMT3B impacts their chromatin targeting remains elusive. This study reveals that the impairment of FF interface by the ICF mutations compromised the heterochromatin association of DNMT3B, consistent with a previous observation that disruption of DNMT3A oligomerization led to its reduced heterochromatin association^10^. Conceivably, the oligomerization of DNMT3A and DNMT3B provides a platform for multivalent protein-protein or protein-DNA interactions between DNMT3A/3B and heterochromatin. On the other hand, the ICF mutations L664P and R670Q, which impair both oligomerization and DNA binding of DNMT3B, may affect the multivalent engagement of DNMT3B within the highly dense heterochromatic environment, leading to reduced heterochromatin association of DNMT3B. Interestingly, the same set of mutations do not seem to severely affect DNMT3B targeting to H3K36me3-demarcated body regions of highly transcribed genes, suggesting that distinctive mechanisms exist mediating the content-dependent targeting of DNMT3B (for instance, heterochromatin vs. body regions of highly transcribed genes). While the molecular basis underlying these distinct mechanisms merits additional investigation, it is possible that the multivalent DNMT3B-protein and DNMT3B-DNA interactions at the H3K9me3- and H3K36me3-enriched regions are differentially affected by the ICF mutations, owing to their different extent of chromatin compaction. In addition, further investigation efforts shall be made towards a more detailed understanding of how various complex states (different degrees of homo- and hetero-oligomerization), protein and DNA bindings, and substrate specificities of DNMT3A and DNMT3B interplay to orchestrate their overlapped but distinct chromatin targeting functions and DNA methylation activities.

## Methods

### Protein expression and purification

The DNA sequences encoding human DNMT3B (NCBI accession NM_006892), full length or the MTase domain (residues 569–853), were inserted into an in-house MBP-tagged expression vector, in which the DNMT3B sequences were separated from an N-terminal hexahistidine (His_6_)-MBP tag by a TEV cleavage site. The *Escherichia coli* BL21 DE3 (RIL) cells transformed with the expression plasmids were first grown at 37 °C, then switched to 16 °C when cell density reached an OD_600_ (optical density at 600 nm) of 1.0, followed by induction with isopropyl β-D-1-thiogalactopyranoside (IPTG). The cells were harvested after overnight growth. The His_6_-MBP-tagged DNMT3B fusion proteins were purified using a Ni^2+^-NTA column. Subsequently, the His_6_-MBP tag was removed through TEV protease cleavage, followed by ion exchange chromatography using a Heparin HP column (GE Healthcare) and further purification through size-exclusion chromatography on a HiLoad 16/600 Superdex 200 pg column (GE Healthcare) in a buffer containing 20 mM Tris-HCl (pH 7.2), 300 mM NaCl, 0.1% β-mercaptoethanol, and 5% glycerol. Purified protein samples were stored at −80°C for future use.

The DNMT3B-DNMT3L complex was prepared as described previously^22^. In essence, the MTase domain of DNMT3B and the C-terminal domain DNMT3L (residues 178-386) were inserted in tandem into a modified PRSF-Duet vector, with the DNMT3B MTase domain preceded by a His_6_-SUMO tag. After co-expression in BL21 DE3 (RIL) cells, the His_6_-SUMO-DNMT3B fusion protein and DNMT3L were first purified using a Ni^2+^-NTA column, followed by removal of the His_6_-SUMO tag via ubiquitin-like-specific protease 1-mediated cleavage. Next, the tag-free DNMT3B-DNMT3L complex was purified sequentially through ion-exchange chromatography and size-exclusion chromatography. The final protein sample of the DNMT3B-DNMT3L complex was stored in a buffer containing 20 mM Tris-HCl (pH 7.2), 100 mM NaCl, 0.1% β-mercaptoethanol, and 5% glycerol. To compare the assembly stability between DNMT3B homo-oligomer and the DNMT3B-DNMT3L complex, the gene fragment encoding residues 178-386 of human DNMT3L (GenBank accession number BC002560) was inserted into pGEX-6P-1 vector with a N-terminal glutathione-S-transferase (GST) tag and 3 C PreScission protease cleavage site. The GST-DNMT3L fusion protein was expressed in *Escherichia coli* BL21 DE3 (RIL) cells, followed by purification using the Glutathione Sepharose 4 Fast Flow column with buffer A (50 mM Tris-HCl (pH 8.0) and 300 mM NaCl) for loading and washing, and buffer B (50 mM Tris-HCl (pH 8.0), 300 mM NaCl and 20 mM glutathione) for elution. Subsequently, the GST tag was cleaved by 3 C PreScission protease and tag-free DNMT3L was purified by GSTrap FF column (GE healthcare). The DNMT3L protein was finally purified by size-exclusion chromatography on a Superdex 200 10/300 GL column (GE Healthcare) equilibrated with buffer containing 25 mM Tris-HCl (pH 7.2), 300 mM NaCl and 1 mM DTT. The purified DNMT3L was concentrated and stored in −80 °C freezer before use.

### Crystallization and structure determination

0.1 mM DNMT3B was mixed with 0.1 mM SAH prior to crystallization. The crystals of DNMT3B were generated by the hanging-drop vapor-diffusion method at 4 °C from drops mixed from 0.5 μL of the protein solution and 0.5 μL of precipitation solution containing 0.1 M MES (pH 6.5), 1.4 M Ammonium sulfate, 0.2 mM SAH and 8% v/v 1,4-Dioxane, and improved by seeding. All crystals were soaked in a cryoprotectant made of the precipitation solution supplemented with 25% glycerol, before flash frozen in liquid nitrogen for X-ray data collection. The X-ray diffraction dataset for DNMT3B was collected on the beamline 24-ID-C, NE-CAT at Advanced Photo Source (APS). The diffraction data were indexed, integrated, and scaled using the XDS program^43^. The structures of the complexes were solved by molecular replacement method using PHASER^44^, with the structure of DNMT3B from DNMT3B-DNMT3L-DNA complex (PDB 6U8P) as a search model. Further modelling was performed using COOT^45^ and subjected to refinement using the PHENIX software package^46^. The same R-free test set was used throughout the refinement. The statistics for data collection and structural refinement is summarized in Supplementary Table 1.

### Cell lines and tissue culture

HEK293 cells were purchased from American Tissue Culture Collection (ATCC, #CRL-1573) and cultured and maintained according to vendor’s manuals. Mouse embryonic stem cells (mESCs) with the triple knockout (TKO) of *Dnmt1*, *Dnmt3a* and *Dnmt3b* (a gift from M. Okano^47^) were plated in the dish pre-coated with 0.1% of gelatin and cultured in the high-glucose DMEM base medium (Invitrogen) supplemented with 15% of heat-inactivated fetal bovine serum (FBS, Invitrogen), 1× non-essential amino acids (Invitrogen), 1 mM L-glutamine, 0.1 mM β-mercaptoethanol and 1000Uml−1 leukaemia inhibitory factor (ESGRO LIF; from Millipore Inc).

*Dnmt3l* knockout (KO) was performed in TKO mESCs by using the CRISPR-Cas9-based gene editing approach. As described before^48^, we conducted the in vitro assembly of the ribonucleoprotein (RNP) complex using the Alt-R CRISPR–Cas9 System (acquired from Integrated DNA Technologies [IDT]), which contained the S.p. HiFi Cas9 Nuclease V3, the trans-activating CRISPR RNA (tracrRNA) with ATTO 550 and a pair of sgRNAs designed to target the murine *Dnmt3l* gene (5’-GAAGGCCTTCCATGATCAAG-3’ and 5’-CCGCAAAGTGAGCTGCACAG-3’, synthesized by IDT). In the presence of electroporation enhancer (acquired from IDT), the in-vitro assembled RNP complex was electroporated into TKO cells. Twenty-four hours post-transduction, ESCs were sorted for the ATTO550 (Cy3) positivity, followed by plating of a single cell into each well of culture plates. *Dnmt3l* KO in TKO cells, which generated ESCs with quadruple knockout of *Dnmt1, Dnmt3a, Dnmt3b* and *Dnmt3l* (QKO), was verified by both western blotting for loss of DNMT3L proteins and direct genomic sequencing of the sgRNA-targeted sites (Supplementary Fig. 6).

As described before^22,26^, QKO ESCs were transfected with the pPyCAGIZ vector (a kind gift of J. Wang, Columbia University) carrying the Flag-tagged DNMT3B, either WT or mutant, by using linear polyethylenimine (PEI, Sigma). Two days post-transfection, cells were subject to drug selection in the medium with 100 µg/mL zeocin (Invitrogen) for 10 days, followed by immunoblotting for DNMT3B to verify its stable expression. HEK293 cells with stable expression of Flag-tagged DNMT3B were generated by the same method. Each month, we examine the cells in culture for possible mycoplasma contamination.

### In vitro DNA methylation assay

For the DNA methylation assay with the C-terminal domains of DNMT3B or DNMT3B-DNMT3L, a 20 μL reaction mixture contained 0.75 µM (GAC)_12_ DNA duplex, 0.3 µM DNMT3B or DNMT3B-DNMT3L tetramer, 2.5 μM *S*-adenosyl-L-[methyl-^3^H] methionine (specific activity 18 Ci/mmol, PerkinElmer) in 50 mM Tris-HCl (pH 8.0), 0.05% β-mercaptoethanol, 5% glycerol and 200 μg/mL BSA. The reactions were carried out in triplicate at 37 °C for 40 min before being quenched by addition of 5 µL of 10 mM nonradioactive SAM. Subsequently, 12.5 µL of reaction mixture was spot on DEAE Filtermat paper (PerkinElmer) and dried. Next, 8 µL of the reaction mixture was spot on Hybond N nylon membrane (GE Healthcare).

For detection, the DEAE paper or Hybond N nylon membrane was washed sequentially with 3 × 5 mL of cold 0.2 M ammonium bicarbonate (pH 8.2), 5 mL of deionized water, and 5 mL of ethanol. The DEAE paper was air dried and transferred to scintillation vials filled with 4, 5 mL of ScintiVerse (Fisher). The radioactivity of tritium was measured using a Beckman LS6500 counter.

### Size-exclusion chromatography of purified DNMT3B proteins

For analysis of the C-terminal domains of DNMT3B and DNMT3B-DNMT3L proteins, purified DNMT3B or DNMT3B-DNMT3L proteins (100 µL of 0.5 mg/mL protein) were loaded onto a Superdex^TM^ 200 Increase 10/300 GL column (GE Healthcare). The flow rate was kept constant at 0.3 mL/min using a running buffer of 25 mM Tris-HCl (pH 7.2), 300 mM NaCl and 1 mM DTT.

For analysis of full-length DNMT3B in mESCs, the QKO cells expressing Flag-tagged WT DNMT3B were resuspended in lysis buffer containing 25 mM Tris-HCl (pH 7.5), 400 mM NaCl, 5% Glycerol, 1X proteinase inhibitor cocktail (Roche) and 100 µg/mL DNase I (Sigma-Aldrich), followed by sonication and centrifugation. The supernatant was mixed with 200 µL anti-Flag M2 beads (Sigma-Aldrich), which was pre-equilibrated with the lysis buffer, for 4 h followed by three times of washes with the same lysis buffer on a rotator at 4 °C. Subsequently, Flag-DNMT3B was eluted from the beads using 3X DYKDDDDK peptides (Thermo Scientific). The Flag-DNMT3B sample was concentrated and analyzed using a Superose 6 Increase 10/300 gl column (Cytiva), pre-equilibrated in 25 mM HEPES (pH 7.5), 400 mM NaCl, 5% Glycerol and 5 mM DTT. The fractions were subjected to SDS-PAGE run and transferred to nitrocellulose membrane. Immunoblotting was carried out using a 1:200 dilution of anti-DNMT3B antibody (G-9, sc-376043, Santa Cruz Biotechnology) followed by a 1:10,000 dilution of Goat anti-mouse IgG conjugated to horseradish peroxidase (Sigma). The blot was exposed using the ChemiDoc Imaging System (BioRad). Images were prepared using Image Lab Software. The size-exclusion chromatography with the Superose 6 10/300 gl column was calibrated using a set of molecular weight markers (GE Healthcare), thyroglobulin (669 kDa), aldolase (158 kDa), conalbumin (75 kDa) and ovalbumin (43 kDa).

### ITC binding assay

ITC measurements were performed using a MicroCal iTC200 instrument (GE Healthcare). Prior to the titration, both peptide and protein samples were subject to overnight dialysis against buffer containing 20 mM Tris-HCl (pH 7.2), 200 mM NaCl, 5% glycerol and 2 mM β-mercaptoethanol. To measure the bindings between DNMT3B proteins and the DNA, 0.2 mM 24-mer DNA duplex (upper strand: 5′-GAC GAC GAC GAC GAC GAC GAC GAC-3′) was titrated with 25 μM WT or mutant DNMT3B at 5 °C with a total of 20 injections with a spacing of 180 s and a reference power of 5 μcal/s. Analyses of all data were performed with MicroCal Origin software, fitted with single-site binding mode. The ITC parameters were summarized in Supplementary Table 2.

### Electrophoretic mobility shift assay (EMSA)

DNA binding assay was conducted using 40 nM (GAC)_12_ DNA duplex, titrated against either WT or mutant DNMT3B MTase domain, with protein concentrations ranged from 0 to 800 nM. The binding mixture was incubated for 30 min in a buffer containing 50 mM Tris-HCl (pH 8.0), 25% Glycerol, 8% Glucose, 2 mM DTT, 0.1 mg/mL BSA. The samples were run on a 3–20% gradient polyacrylamide gel at 100 V using 0.5x TBE (pH 8.3) buffer, which lasted 1 h at 4 °C. The gel was stained using SYBR gold stain for visualization.

### Immunofluorescence (IF)

Cells were fixed in 4% of paraformaldehyde for 10 min at room temperature, followed by incubation in 1× PBS containing 0.2% of Triton X-100 for 10 min to permeabilize the cells. Fixed cells were co-stained with primary antibodies. Anti-Flag antibody (F1804, Sigma) was used for Flag-tagged DNMT3B Isoform 1 (DNMT3B1) and rabbit antibodies were used against H3K9me3 (ab8898, Abcam) or H3K36me3 (ab9050, Abcam), followed by staining with the appropriate conjugated secondary antibodies for mouse and rabbit antibodies (which are conjugated with Alexa-488 and Alexa-594, respectively; acquired from Life Technologies). Nuclei were finally stained with 4,6-diamino-2-phenylindole (DAPI, 0.1 g/ml). Images were taken with an FV1000 confocal microscope (Olympus; available at UNC Imaging Core). Signal colocalization analyses were performed using EzColocalization plugin of FIJI^49^. More than 80 cells of each sample were used for IF signal colocalization analysis of DNMT3B1 and H3K9me3, and more than 60 cells used for DNMT3B1 and H3K36me3.

### Co-immunoprecipitation (CoIP)

Briefly, whole cell lysate was prepared in the lysis buffer (50 mM Tris–HCl pH 8.0, 150 mM NaCl, 1% NP-40 and 1× protease inhibitor cocktail freshly added), followed by incubation on ice for 20 min and centrifugation at 10,000 *g* at 4 °C for 10 min. The supernatant was collected and incubated with primary antibodies against protein of interest on a rotator overnight in a cold room, followed by addition of protein A/G beads for additional 2 h. Beads were washed three times in the lysis buffer, and then analyzed by immunoblotting.

### Chromatin immunoprecipitation (ChIP) followed by quantitative PCR (ChIP-qPCR)

ChIP-qPCR was conducted following a previously published protocol^47,50^. In brief, 10 million of cells with the stable expression of Flag-DNMT3B1 were rinsed in the ice-cold PBS twice and cross-linked with 1% of formaldehyde at room temperature for 10 min, followed by addition of glycine to quench crosslinking. Cells were collected and re-suspended, followed by sonication by using a Bioruptor (Diagenode). After centrifugation, the cleared chromatin sample was harvested and incubated with anti-Flag (F1804, Sigma) antibody-conjugated Dynabeads (Invitrogen) at 4 degree for overnight. Beads bound with chromatin were subject to extensive washing, followed by elution, de-crosslinking overnight at 65 degree, and sequential digestion by proteinase K and RNase A to remove protein and RNA. The genomic DNA sample from the ChIP experiment was finally purified, diluted and used for ChIP-qPCR using the primers specific for genomic site of interest. ChIP signals, generated from at least three independent experiments, were normalized to those of input sample and presented as mean ± SD. Student’s *t*-test was used to ascertain signal difference. The sequence information of primers used for ChIP-qPCR is summarized in Supplementary Table 3.

### Quantifications of 5-methyl-2′-deoxycytidine (5-mdC) and 2′-deoxyguanosine (dG) in genomic DNA by using LC-MS/MS/MS

The measurement of 5-mdC and dG in genomic DNA followed a previously published protocol^22,26^. Briefly, 1 μg of genomic DNA was digested with 0.1 unit nuclease P1 in a buffer containing 30 mM sodium acetate (pH 5.6) and 1 mM ZnCl_2_. After incubation at 37 °C for 24 h, 0.5 unit of alkaline phosphatase and 0.001 unit of phosphodiesterase I in 50 mM Tris-HCl (pH 8.6) were added to the digestion mixture. The mixture was incubated at 37 °C for another 2 h. [^13^C_5_]−5-mdC and [^15^N_5_]-dG were added to the enzymatic digestion mixture of 50 ng of DNA. The enzymes in the digestion mixture were removed by chloroform extraction. The resulting aqueous layer was dried by using a Speed-vac and the dried residues were subsequently reconstituted in doubly distilled water. The digestion mixture of ~ 5 ng DNA was subjected to LC-MS/MS/MS analyses for the quantifications of 5mdC and dG. An LTQ XL linear ion trap mass spectrometer (Thermo Fisher Scientific) equipped with a nanoelectrospray ionization source and coupled with an EASY-nLC II system (Thermo Fisher Scientific) was used to perform the LC-MS/MS/MS experiments. The amounts of 5-mdC and dG (in moles) in the nucleoside mixtures were calculated from area ratios of peaks found in selected-ion chromatograms (SICs) for the analytes over their corresponding isotope-labeled standards, the amounts of the labeled standards spiked in (in moles), and the calibration curves (Supplementary Data 1–5). The final levels of 5-mdC, in terms of percentages of dG, were calculated by dividing the molar quantities of 5-mdC with those of dG.

### Statistics and reproducibility

The two-tailed Student *t*-test was performed to compare distributions between different groups. And the *p* value lower than 0.05 was considered to be statistically significant. The statistical methods used for analysis of ChIP-qPCR and Bisulfite sequencing results are described in the above sections. Experiments such as EMSA and immunoblotting were repeated at least twice and those of ChIP-qPCR at least three times.

## Supplementary information

Supplementary information

Supplementary Data

## Data Availability

The coordinate and structure factor for the homo-oligomeric DNMT3B [https://www.ncbi.nlm.nih.gov/nuccore/NM_006892] have been deposited in the Protein Data Bank under accession code 7V0E. The biochemical and cellular data generated in this study are provided in the Supplementary Information/Supplementary Data/Source Data file. Source data are provided with this paper.

## Source data

Source data file
