# Supplementary material for: Structure of DNMT3B homo-oligomer reveals vulnerability to impairment by ICF mutations
Source: Nat Commun. 2022 Jul 22;13:4249. doi: 10.1038/s41467-022-31933-w (PMC9307851; doi:10.1038/s41467-022-31933-w)
Supplement: Supplementary file 1 — Supplementary information [file 41467_2022_31933_MOESM1_ESM.pdf]

Supplementary information

Structure of DNMT3B homo-oligomer reveals vulnerability to impairment by ICF mutations

Linfeng Gao, Yiran Guo, Mahamaya Biswal, Jiuwei Lu, Jiekai Yin, Jian Fang, Xinyi Chen, Zengyu Shao, Mengjiang Huang, Yinsheng Wang, Gang Greg Wang, Jikui Song

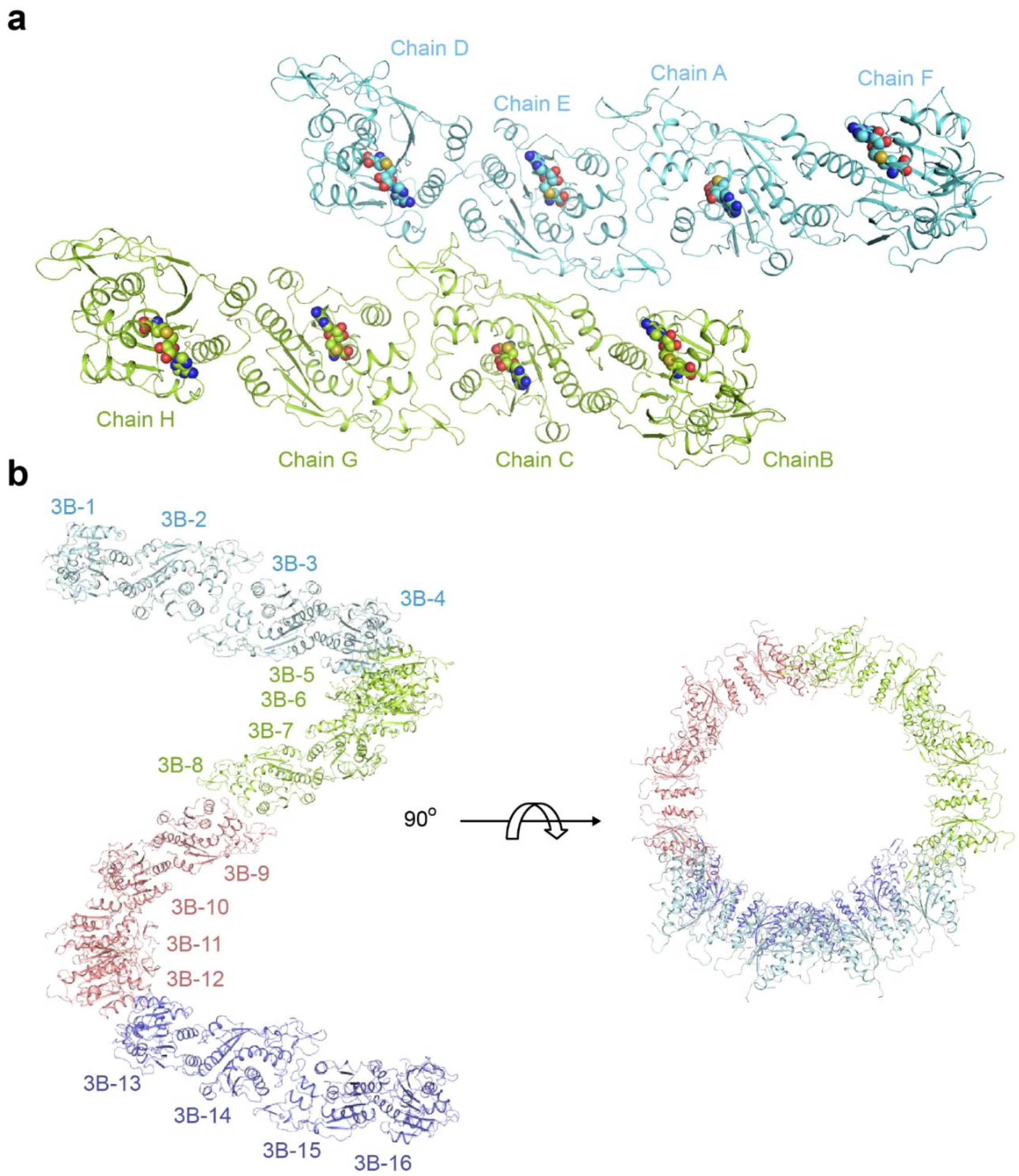

Supplementary Figure 1. Analysis of DNMT3B oligomerization in the crystal. (a) The DNMT3B oligomers in one asymmetric unit. Chains B, C, G and H were chosen for structural analysis. (b) DNMT3B molecules were packed into a left-handed helical structure in the crystal with a decamer as the repeating unit. The individual tetramers are color coded.

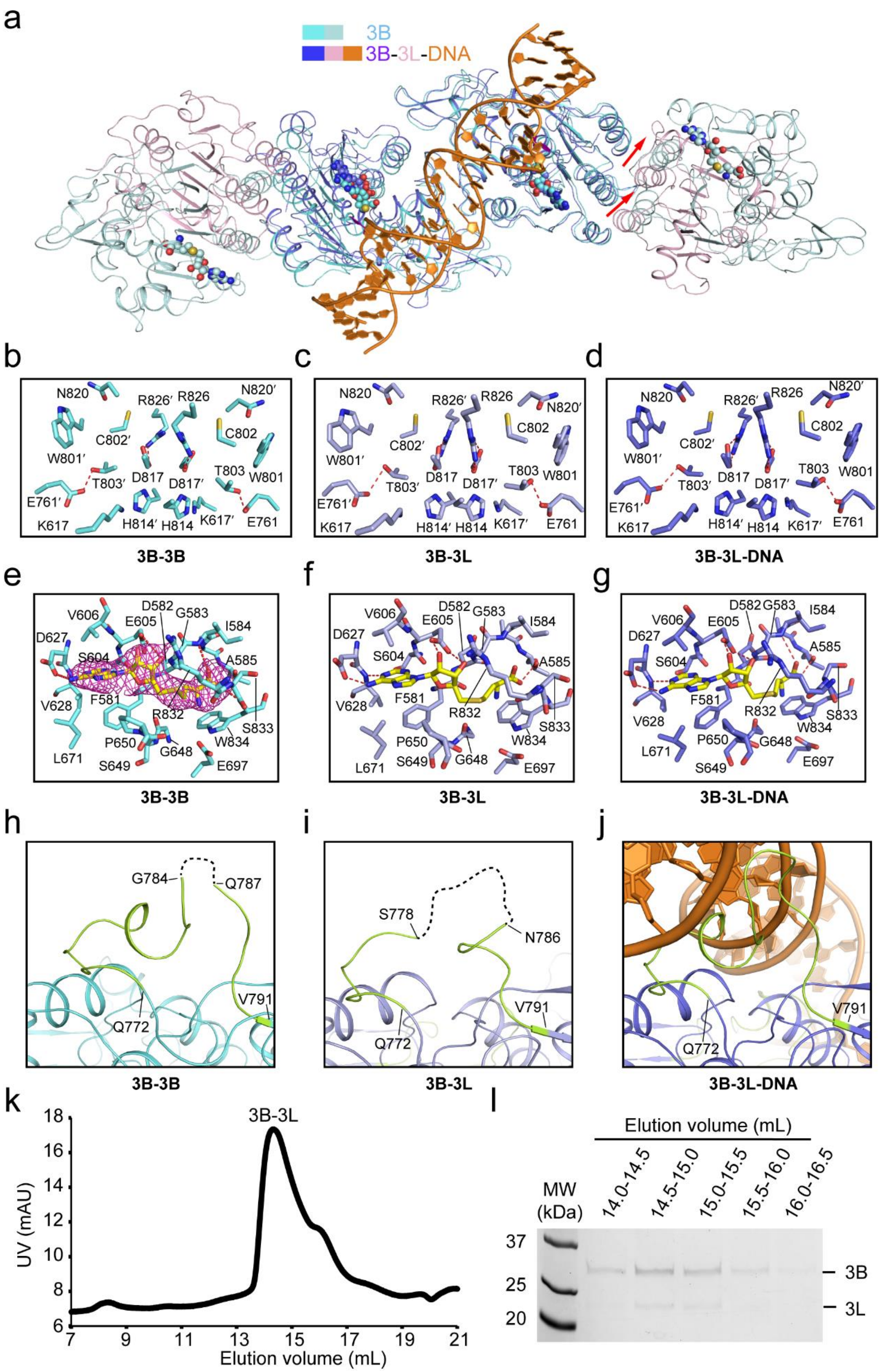
a
3B
3B-3L-DNA
b
N820
R826′
R826
N820′
C802′
C802
W801′
T803
W801
T803′
D817
D817′
E761′
K617
H814′
H814
K617′
E761
3B-3B
c
3B-3L
d
3B-3L-DNA
e
V606
D582
G583
E605
I584
D627
S604
A585
V628
F581
R832
S833
P650
W834
G648
L671
S649
E697
3B-3B
f
3B-3L
g
3B-3L-DNA
h
G784
Q787
Q772
V791
3B-3B
i
S778
N786
Q772
V791
3B-3L
j
Q772
V791
3B-3L-DNA
k
3B-3L
UV (mAU)
Elution volume (mL)
l
Elution volume (mL)
MW (kDa)
14.0-14.5
14.5-15.0
15.0-15.5
15.5-16.0
16.0-16.5
37
25
20
3B
3L

Supplementary Figure 2. Additional details for the structural comparison between DNMT3B-DNMT3B and DNMT3B-DNMT3L complexes. (a) Structural overlay between the DNMT3B-DNMT3B and DNMT3B-DNMT3L-DNA (PDB 6U8P) complexes. The lateral shift in helical packings at the FF interface between the two complexes is indicated by red arrows. (b-d) Close-up views of the RD interface in the DNMT3B-DNMT3B (b), DNMT3B-DNMT3L (PDB 6KDP) (c) and DNMT3B-DNMT3L-DNA (PDB 6U8P) complexes. The hydrogen bonds are shown as dashed lines. (e-g) Close-up views of the SAH (yellow stick) binding in the DNMT3B-DNMT3B (e), DNMT3B-DNMT3L (PDB 6KDP) (f) and DNMT3B-DNMT3L-DNA (PDB 6U8P) complexes (g). The hydrogen bonds are shown as dashed lines. The Fo-Fc omit map (2σ contour) for the bound SAH molecule is shown as magenta mesh in e. (h-j) Close-up views of the TRD loop (limon; residues Q772-V791) in the DNMT3B-DNMT3B (h), DNMT3B-DNMT3L (PDB 6KDP) (i) and DNMT3B-DNMT3L-DNA (PDB 6U8P) complexes (j). The disordered regions in (h) and (i) were depicted as dashed lines. (k) Size exclusion chromatography of DNMT3B MTase protein sample mixed with the DNMT3L C-terminal domain in a 1:1 molar ratio on a Superdex 200 Increase 10/300 gl column. (l) SDS-PAGE images of the fractions collected from (k). The protein bands corresponding to DNMT3B and DNMT3L C-terminal domains are indicated. The experiment in (k-l) was performed twice with consistent results. Source data are provided as a Source Data file.

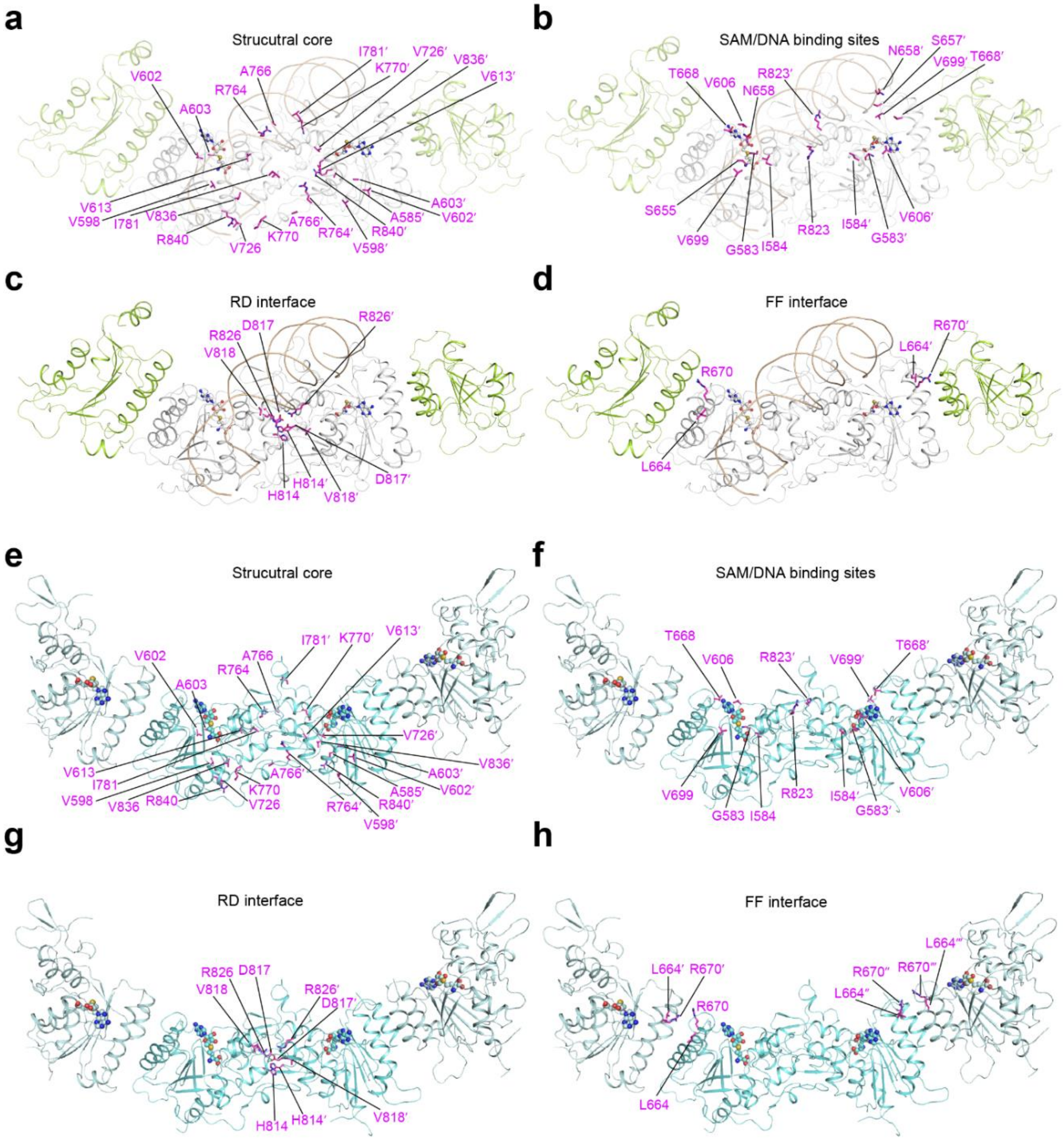

Supplementary Figure 3. Structural mapping of the ICF mutations. (a-d) Structural mapping of the ICF mutations onto the structure of the DNMT3B-DNMT3L-DNA complex (PDB 6U8P), classified into individual regions, including the structure core (a), DNA- and cofactor-binding sites (b), RD interface (c), and FF interface (d). (e-h) Structural mapping of the ICF mutations onto the structure of the DNMT3B-DNMT3B complex, classified into

individual regions, including the structure core (e), DNA- and cofactor-binding sites (f), RD interface (g), and FF interface (h). The mutation sites are shown in stick representation and labeled.

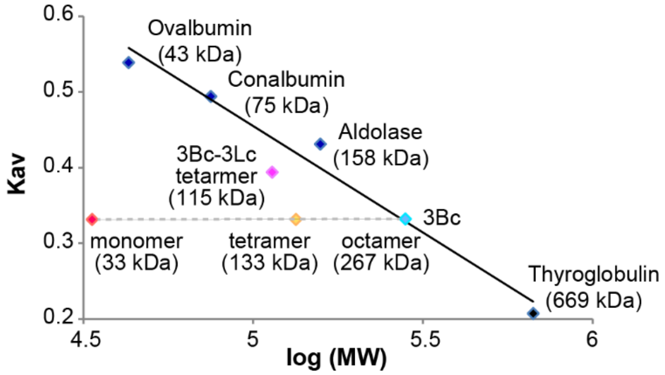

Supplementary Figure 4. Size-exclusion chromatography analysis of the C-terminal domains of DNMT3B-DNMT3B (3Bc) and DNMT3B-DNMT3L (3Bc-3Lc) complexes. The Superdex 200 Increase 10/300 gl column was calibrated with Thyroglobulin, Aldolase, Conalbumin, and Ovalbumin proteins. The calibration curve was obtained by fitting the gel phase distribution coefficient (Kav) with the logarithm of molecular weight, log (MW). The elution volume of each protein was used to calculate the Kav using the formula: Kav = (Ve - Vo)/(Vc – Vo), where Ve= elution volume of protein, Vo= column void volume, Vc= geomtric column volume. The Thyroglobulin, Aldolase, Conalbumin, and Ovalbumin proteins are marked as blue diamonds in accordance with their respective coordinates of Kav and log (MW). The predicted coordinates for the tetrameric and/or other assembly forms of DNMT3B-DNMT3B or DNMT3B-DNMT3L are marked as magenta (3Bc-3Lc tetramer), red (3Bc monomer), yellow (3Bc tetramer) or cyan (3Bc octamer) diamonds. This data supports the oligomeric assemblies of 3Bc-3Lc and 3Bc-3Bc in solution.

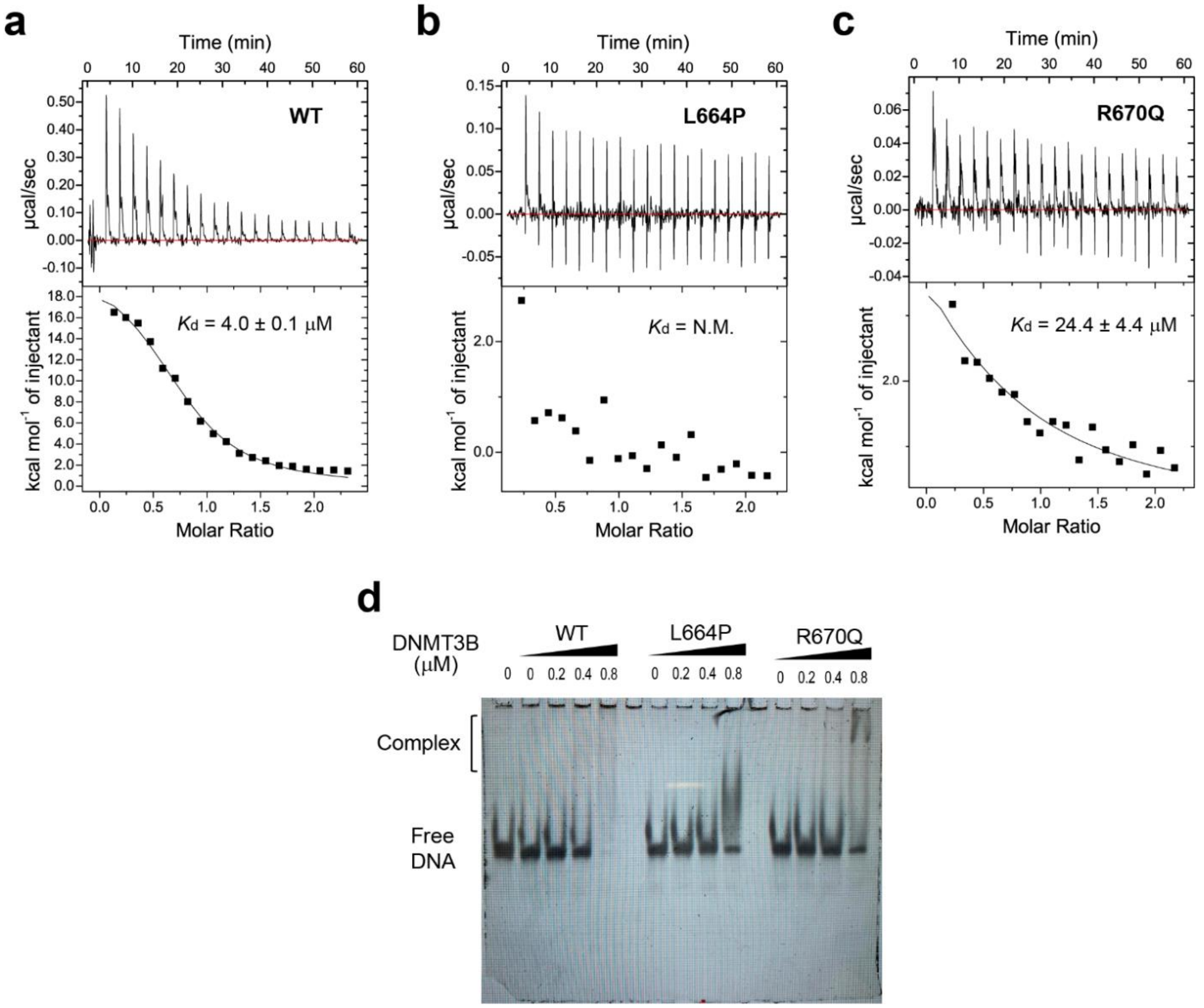

Supplementary Figure 5. Impact of the ICF mutations on the FF interface on the DNA-binding activity of DNMT3B. (a-c) ITC binding assays for the MTase domain of wild-type (WT) DNMT3B (a), L668P-mutated DNMT3B (b), and R670Q-mutated DNMT3B (c) over a 24-mer DNA duplex containing multiple CG sites. N.M., not measurable. The experiment for (a) was performed twice with consistent results. The experiment for (b) or (c) was performed once. (d) EMSA analysis of WT and mutant DNMT3B with the increasing concentration of DNMT3B molecules. The experiment was repeated twice with consistent results. Source data are provided as a Source Data file.

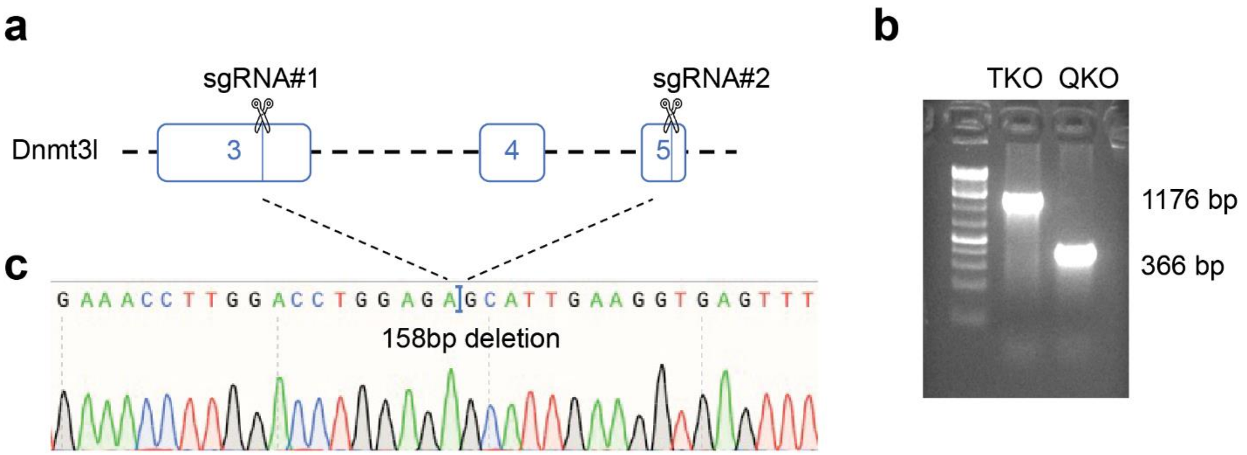

Supplementary Fig 6. CRISPR/cas9-mediated Dnmt3l knockout (KO) in TKO mESCs, producing QKO. (a) Schematic of the CRISPR/cas9-mediated Dnmt3l KO. A pair of guide RNAs were designed to target Dnmt3l exons 3 and 5 respectively, with the site of the first sgRNA (sgRNA#1) approximately 58 bp downstream of the start codon in exon 3. (b) After CRISPR/cas9-mediated editing, PCR was conducted with genomic DNA and a pair of genotyping primers flanking the edited region, which identified a clonal mESC line harboring the homozygous genomic deletion at Dnmt3l (QKO; right lane). Parental TKO mESCs (left lane) serve as control. The experiment was performed once. Source data are provided as a Source Data file. (c) Sanger sequencing results using the above genomic PCR product verified homozygous genomic deletion of Dnmt3l, which causes the frame-shift (due to a 158 bp deletion of its coding sequence from exons 3-5) and disruption of Dnmt3l.

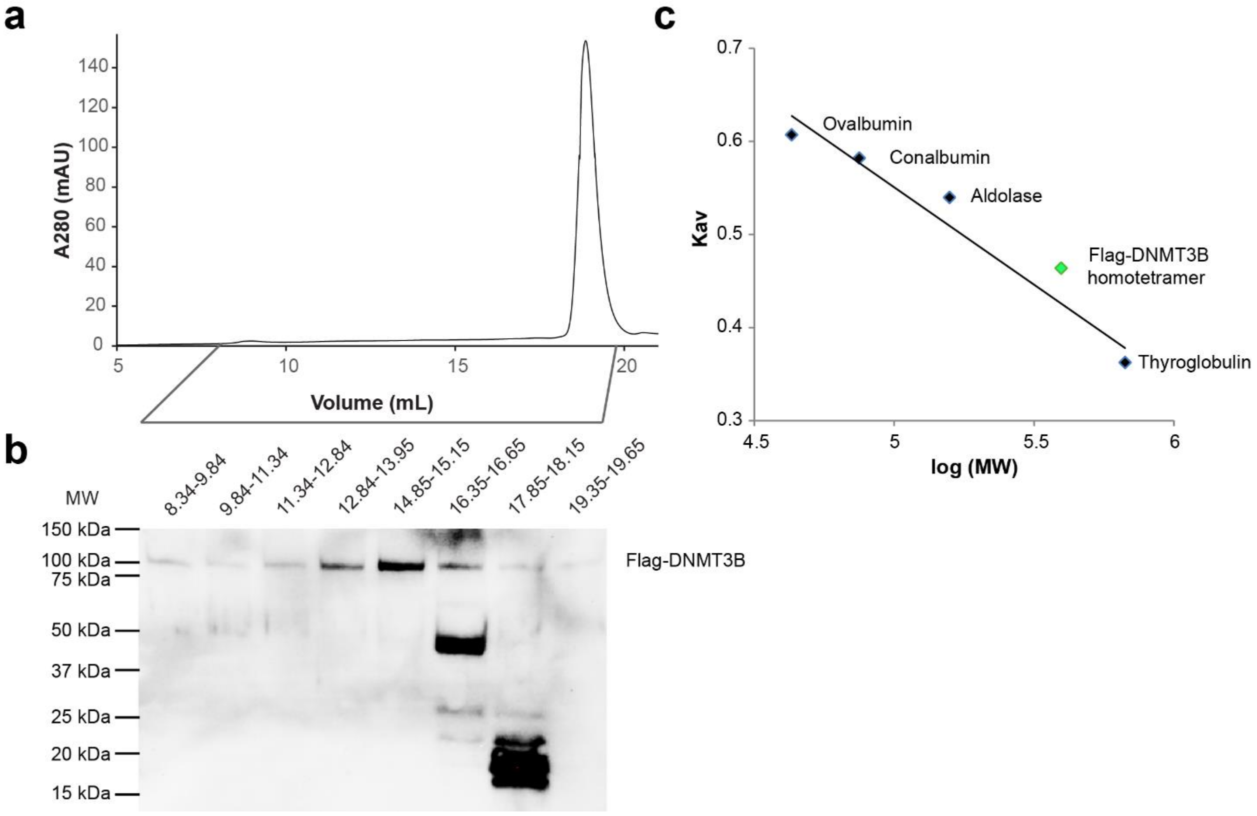

Supplementary Figure 7. Size-exclusion chromatography analysis of the Flag-tagged, full-length WT DNMT3B protein purified from the DNMT3B-transfected QKO cells. (a) The elution profile of Flag-DNMT3B on a Superose 6 10/300 column. (b) The western blot images for the fractions collected from (a). The experiment was performed once. Source data are provided as a Source Data file. (c) The Superose 6 10/300 column was calibrated with Thyroglobulin, Aldolase, Conalbumin, and Ovalbumin proteins. The thyroglobulin, aldolase, conalbumin, and ovalbumin proteins are marked as blue diamonds in accordance with their respective coordinates of Kav and log (MW). The predicted coordinate for the tetrameric form of Flag-DNMT3B is marked as a blue diamond.

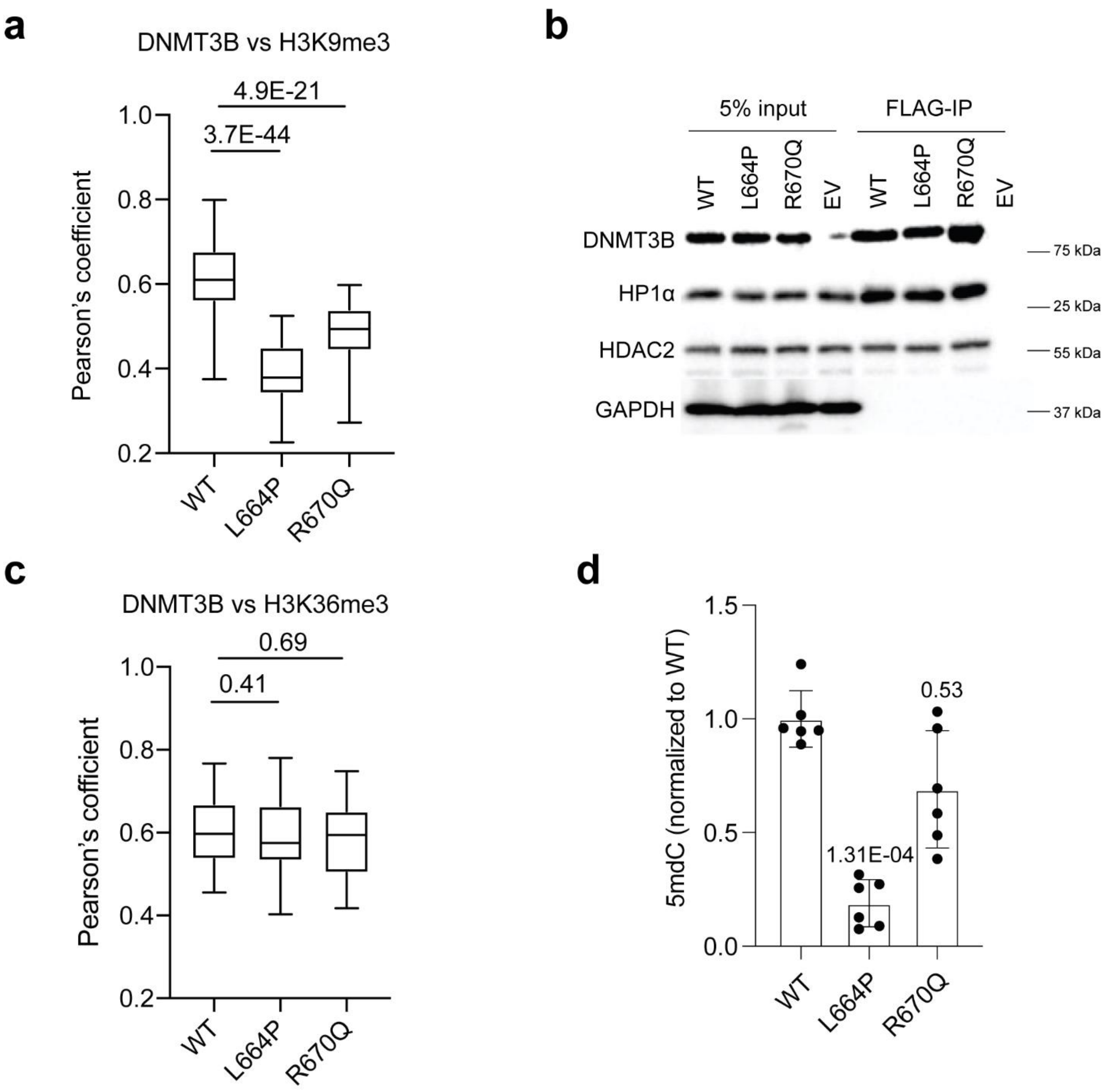

Supplementary Fig 8. Effect of the FF-interface mutations on genomic localization and DNA methylation of DNMT3B. (a) Quantification of DNMT3B and H3K9me3 co-localization, based on their IF signals in QKO ESCs with expression of the indicated DNMT3B, either WT (n = 92) or the mutant of L664P (n = 84) or R670Q (n = 87). The box plot shows the maximum, the 75th percentile, median, 25th percentile and minimum values. (b) Co-immunoprecipitation (CoIP) for interaction of HP1α and HDAC2 with Flag-tagged DNMT3B, either WT or the indicated mutant expressed in in HEK293 cells. EV, empty vector control. The experiment was repeated twice with consistent results. Source

data are provided as a Source Data file. (c) Quantification of DNMT3B and H3K36me3 co-localization, based on their IF signals in QKO ESCs with expression of the indicated DNMT3B, either WT (n = 68) or the mutant of L664P (n = 86) or R670Q (n = 62). The box plot shows the maximum, the 75th percentile, median, 25th percentile and minimum values. (d) Mass spectrometry-based quantification for global DNA methylation levels in TKO cells with stable expression of the indicated DNMT3B (n = 6). The two-tailed Student t-test was applied for statistics of mutant vs WT, with p-values labeled on the top of each panel. Source data are provided as a Source Data file.

Supplementary Table 1. Data collection and refinement statistics

	DNMT3B (PDB 7V0E)
**Data collection**	
Space group	$P\ 3_2$
Cell dimensions	
a, b, c (Å)	164.4, 164.4, 209.2
α, β, γ (°)	90, 90, 120
Resolution (Å)	142.37-3.27 (3.33-3.27)[a]
R_{merge}	0.160 (0.983)
$I/\sigma(I)$	8.2 (1.3)
$CC_{1/2}$	0.980 (0.360)
Completeness (%)	95.3 (91.5)
Redundancy	3.0 (2.9)
**Refinement**	
Resolution (Å)	84.29-3.27 (3.39-3.27)
No. reflections	93089 (8881)
R_{work} / R_{free}	20.6/23.5 (34.1/39.6)
No. atoms	
Protein	17023
SAH	208
Water	29
B factors (Å^2)	
Protein	67.8
SAH	58.5
Water	48.58
R.m.s. deviations	
Bond lengths (Å)	0.007
Bond angles (°)	0.868

[a]Values in parentheses are for highest-resolution shell.

Supplementary Table 2. Summary of ITC binding parameters

**Protein**	**K_d (μM)**	**N value**
WT	4.0 ± 0.1	0.61 ±0.23
R670Q*	24.4 ± 4.4	0.5
L664P	N.M.	

For WT, the mean value and S.D. were derived from two independent measurements. For R670Q and L664P, the parameters were derived from single measurement. *For R670Q, the N value was manually set to 0.5, assuming one DNA molecule binds to two DNMT3B molecules. N.M., not measurable.

Supplementary Table 3. Reagents used in this study

**Primers used for ChIP-qPCR**		
Name	Forward	Reverse
Major satellite set 1 (P1)	GATTTCGTCATTTTTCAAGTCGTC	TTTAGAAATGTCCACTGTAGG
Major satellite set 2 (P2)	GACGACTTGAAAAATGACGAAATC	CATATTCCAGGTCCTTCAGTGTGC
Minor satellite set 1 (P1)	ACTCATCTAATATGTTCTACAGTG	AAAACACATTCGTTGGAAACGGG
Minor satellite set 2 (P2)	CAT GGAAAATGATAAAAACC	CATCTAATATGTTCTACAGTGTGG
Zfp777_5exon	GTCTCAAAACAGACTCCACC	CCCAGCCTAGCAAAGAAATT
Ankrd10_3exon	GCTATGAAGCTTTGGGTCAA	GGATTAGAGGAGAGTGCCTT
LINE1	AACCTACTTGGTCAGGATGGATG	AGTGCAGAGTTCTATCAGACCTTC
IAP 5'-LTR	CTCCATGTGCTCTGCCTTCC	CCCCGTCCCTTTTTTAGGAGA
**Primers used for bisulfite sequencing studies**		
Name	Forward	Reverse
IAP 5'-LTR	YTGAYAGYTGTGTTYTAAGTGGTA AATAAA	ARAACACCACARACCARAATCTTCT RC
**Primers used for biochemical characterizations**		
Name	Forward	Reverse
WT	GTACATCCAAGGATCCGCAGCCCG AAGGCGG	GTCCTACAGGCGCGCCTTATTCA CATGCAAAGTAGTCCTTCA
L664P	CAGGAAAGGCCCGTATGAGGGTAC AGGCCGG	CTGTACCCTCATACGGGCCTTTCCTG GCTGGATTCA
L664T	CAGGAAAGGCACGTATGAGGGTAC AGGCCG G	CTGTACCCTCATACGTGCCTTTCCTG GCTGGATTCA
R670Q	GTATGAGGGTACAGGCCAGCTCTT CTTCGAATTTTACC ACCTGCTGA	GTAAAATTCGAAGAAGAGCTGGCCT GTACCCTCATACAGGCCTTTC

**Antibodies**			
Name	Vendor	Catalog no.	Usage
anti-DNMT3B	Santa Cruz	sc-376043	WB
anti-FLAG	Sigma-Aldrich	F1804	WB,; ChIP-qPCR; IF
anti-H3K9me3	Abcam	ab8898	IF
anto-H3K36me3	Abcam	ab9050	IF
anti-GAPDH	Cell Signaling	#5174	WB
anti-DNMT3L	Cell Signaling	#13451	WB
