# Supplementary material for: Structure of DNMT3B homo-oligomer reveals vulnerability to impairment by ICF mutations
Source: Nat Commun. 2022 Jul 22;13:4249. doi: 10.1038/s41467-022-31933-w (PMC9307851; doi:10.1038/s41467-022-31933-w)

Supplementary Data 1. LC-MS/MS quantification of 5-mdC in the nucleoside mixture arising from the digestion of DNA isolated from QKO cells with stable expression of WT DNMT3B. (a) Representative selected-ion chromatograms (SICs) for monitoring the *m/z* 242→126→109 and 247→126→109 transitions for the $[M + H]^+$ ions of 5-mdC and [$^{13}C_5$]-5-mdC, respectively. (b) Representative SICs for monitoring the *m/z* 268→152→135 and 273→157→139 transitions for the $[M + H]^+$ ions of dG and [$^{15}N_5$]-dG, respectively.

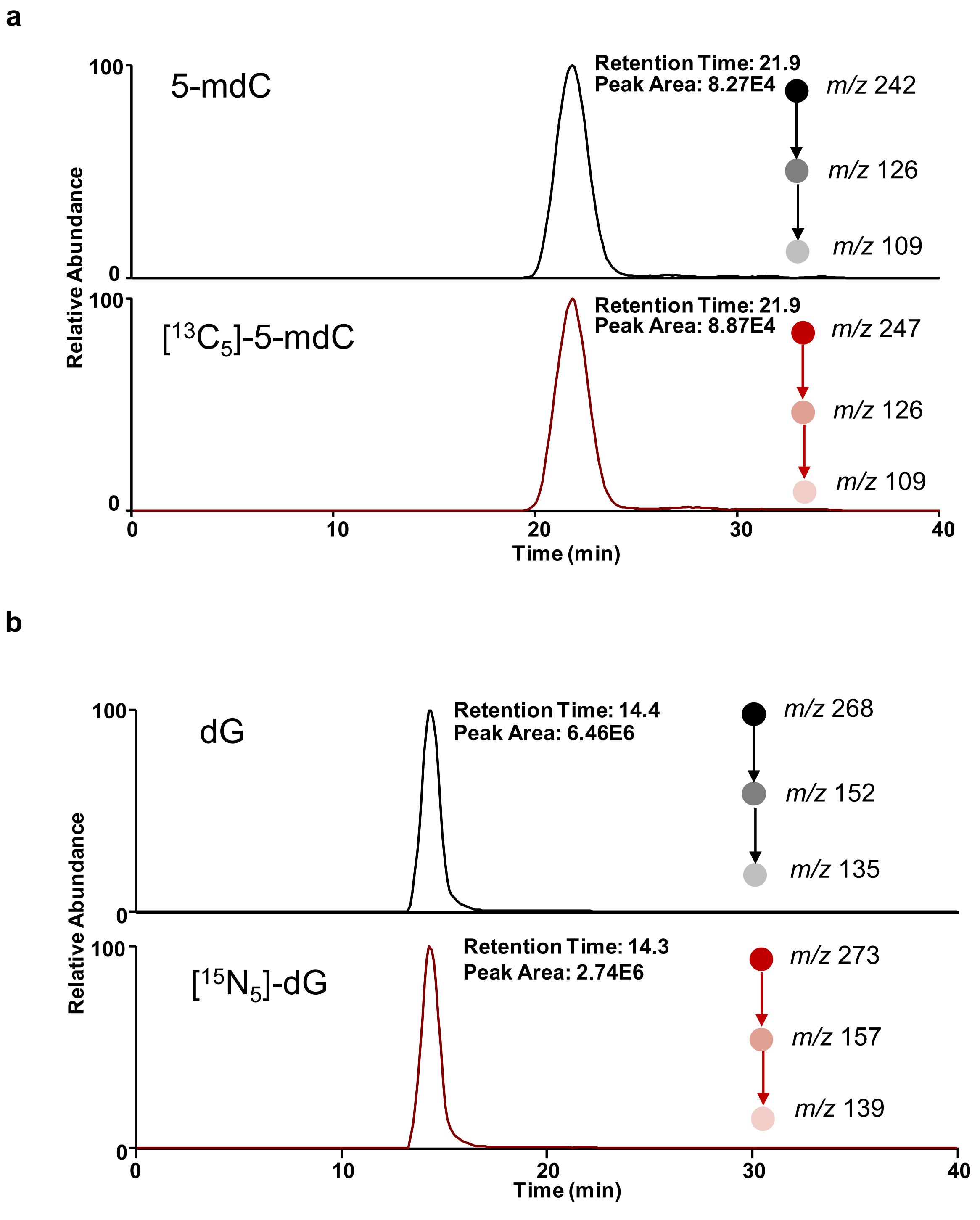

Supplementary Data 2. Positive-ion ESI-MS/MS/MS for monitoring the further fragmentation of the $[M+H]^+$ ions of the nucleobase portions of 5-mdC (*m/z* 242$\rightarrow$126$\rightarrow$, a), $[^{13}C_5]$-5-mdC (*m/z* 247$\rightarrow$126$\rightarrow$, b), dG (*m/z* 268$\rightarrow$152$\rightarrow$, c), and $[^{15}N_5]$-dG (*m/z* 273$\rightarrow$157$\rightarrow$, d).

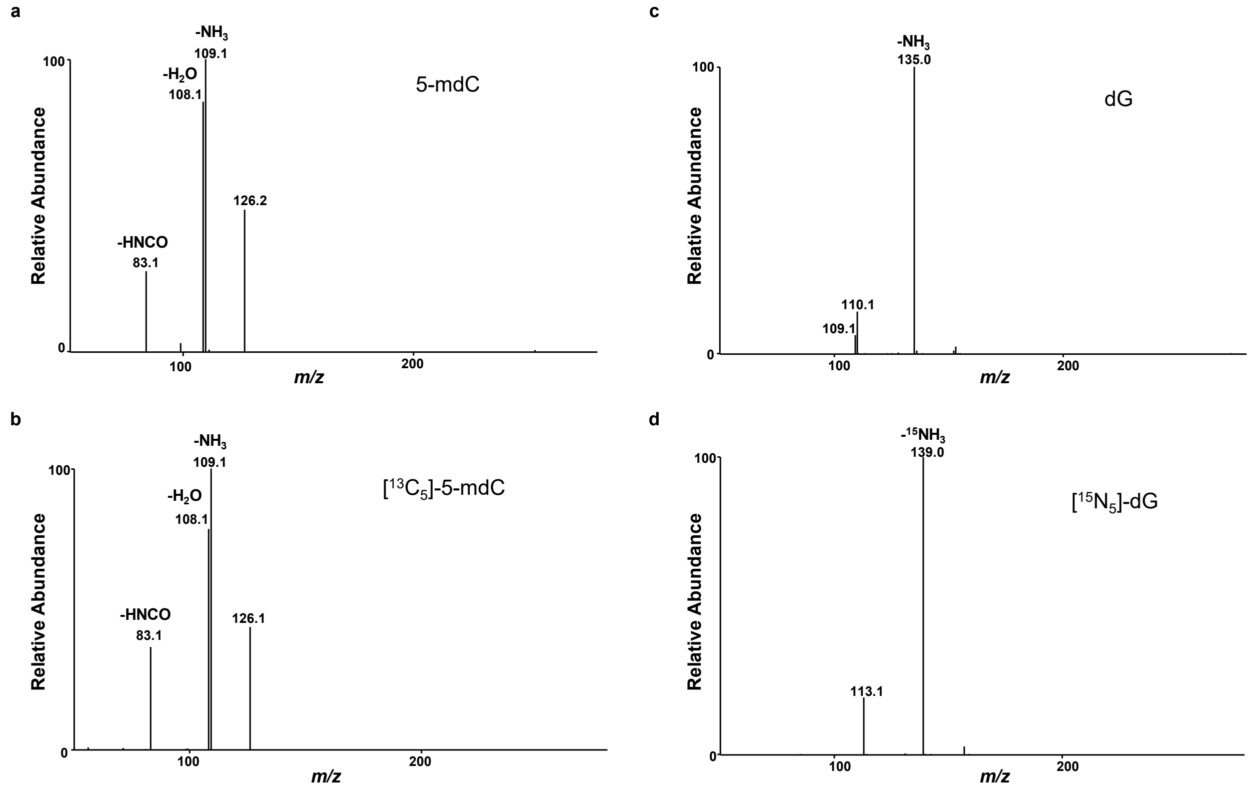

Supplementary Data 3. LC-MS/MS quantification of 5-mdC in the nucleoside mixture arising from the digestion of DNA isolated from QKO mouse ES cells. (a) Representative SICs for monitoring the *m/z* 242→126→109 and 247→126→109 transitions for the $[M + H]^+$ ions of 5-mdC and [$^{13}C_5$]-5-mdC, respectively. (b) Representative SICs for monitoring the *m/z* 268→152→135 and 273→157→139 transitions for the $[M + H]^+$ ions of dG and [$^{15}N_5$]-dG, respectively.

a

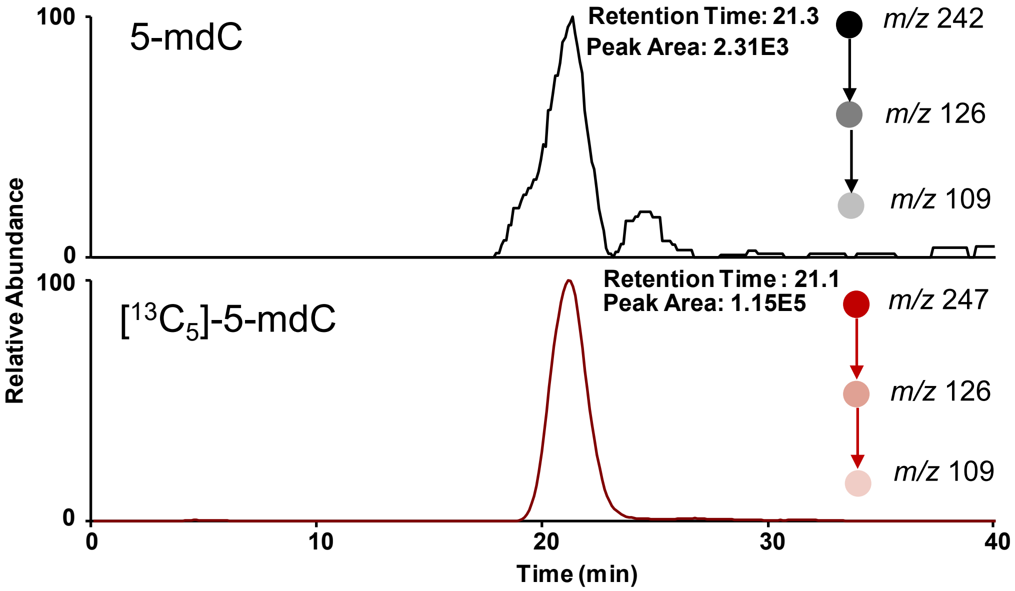

b

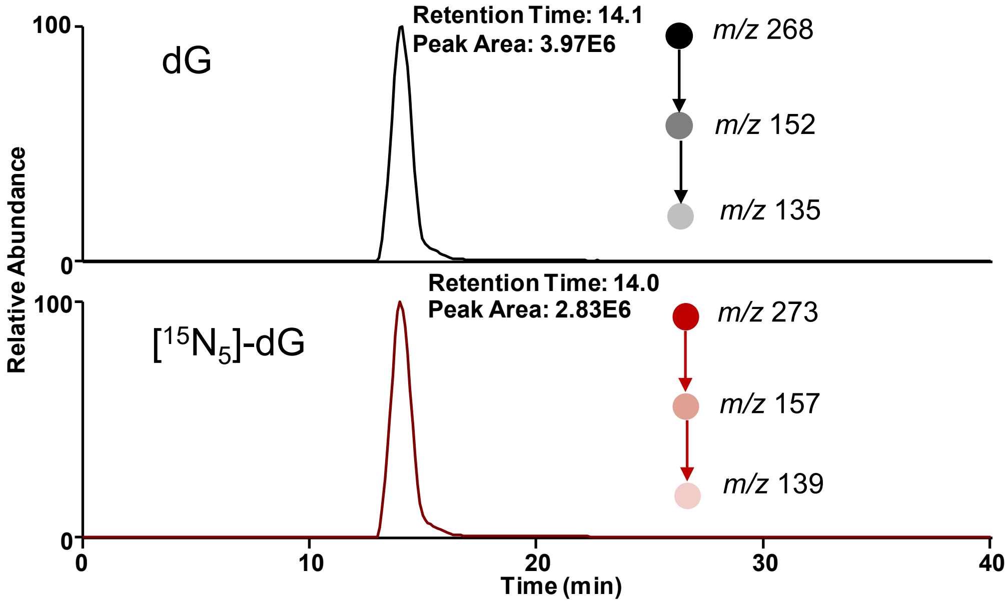

Supplementary Data 4. LC-MS/MS quantification of 5-mdC in the nucleoside mixture arising from the digestion of DNA isolated from QKO cells with stable expression of L664P DNMT3B. (a) Representative SICs for monitoring the *m/z* 242→126→109 and 247→126→109 transitions for the $[M + H]^+$ ions of 5-mdC and [$^{13}C_5$]-5-mdC, respectively. (b) Representative SICs for monitoring the *m/z* 268→152→135 and 273→157→139 transitions for the $[M + H]^+$ ions of dG and [$^{15}N_5$]-dG, respectively.

a

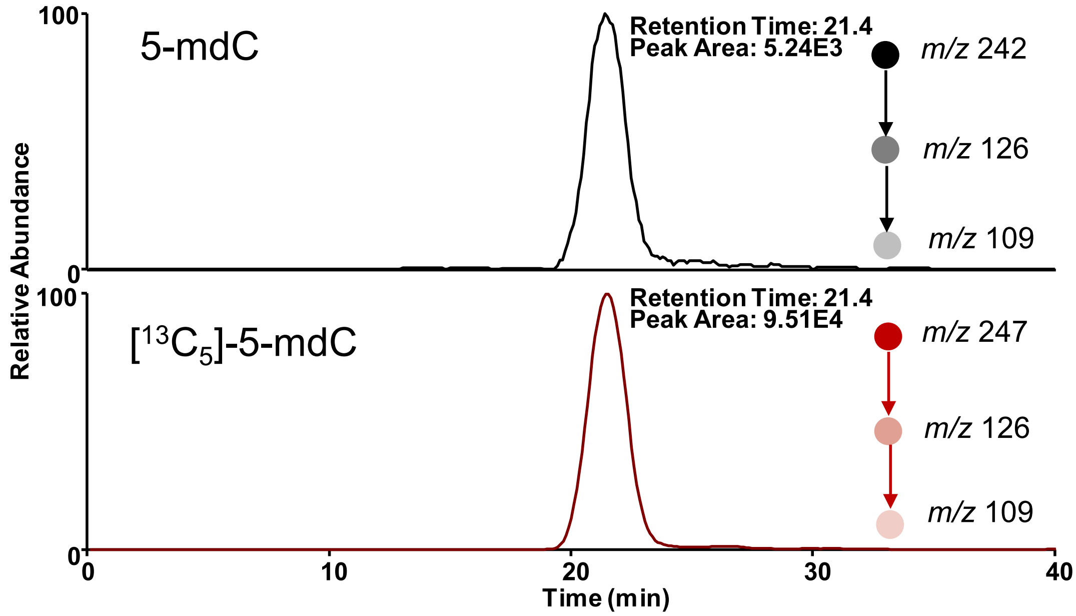

b

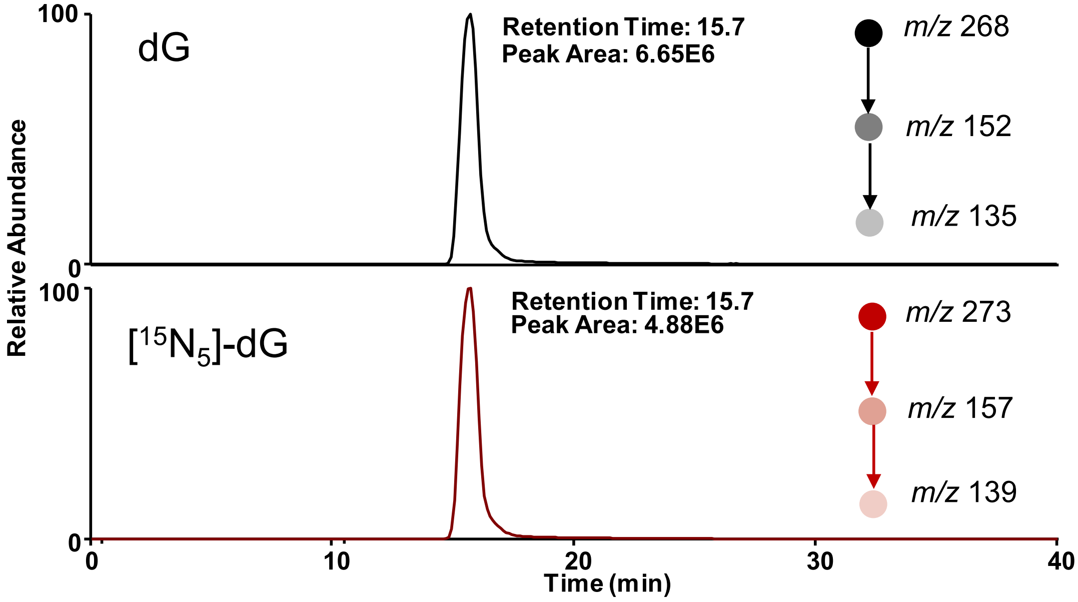

Supplementary Data 5. LC-MS/MS quantification of 5-mdC in the nucleoside mixture arising from the digestion of DNA isolated from QKO cells with stable expression of R670Q DNMT3B. (a) Representative SICs for monitoring the *m/z* 242→126→109 and 247→126→109 transitions for the $[M + H]^+$ ions of 5-mdC and [$^{13}C_5$]-5-mdC, respectively. (b) Representative SICs for monitoring the *m/z* 268→152→135 and 273→157→139 transitions for the $[M + H]^+$ ions of dG and [$^{15}N_5$]-dG, respectively.

a

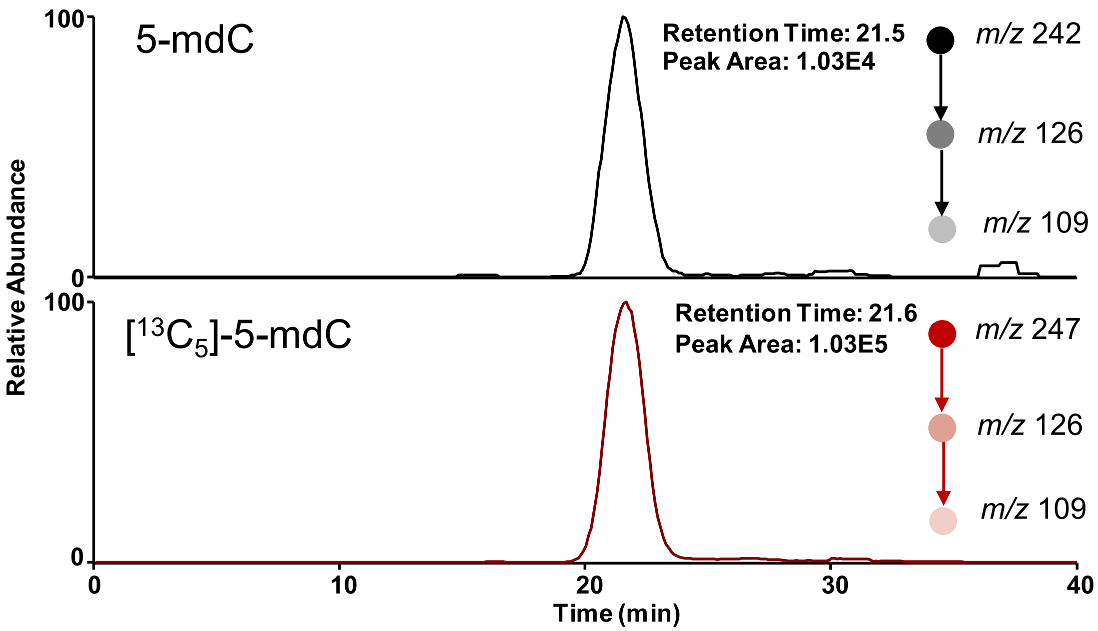

b

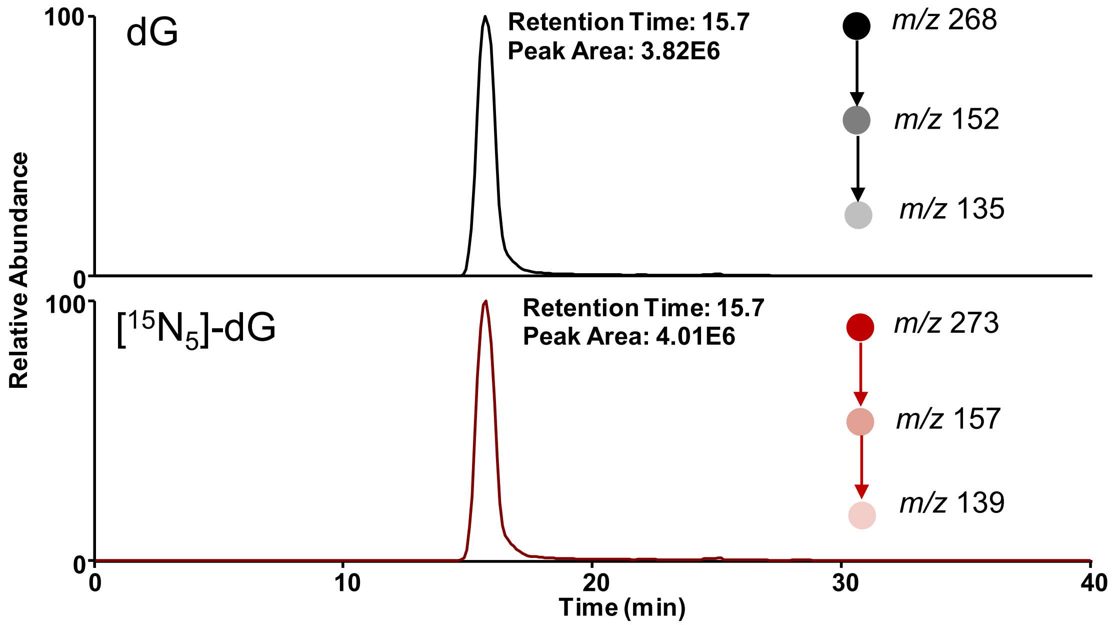

Supplement: Supplementary file 2 — Supplementary Data [file 41467_2022_31933_MOESM2_ESM.pdf]
